# A Bayesian model selection approach to mediation analysis

**DOI:** 10.1371/journal.pgen.1010184

**Published:** 2022-05-09

**Authors:** Wesley L. Crouse, Gregory R. Keele, Madeleine S. Gastonguay, Gary A. Churchill, William Valdar

**Affiliations:** 1 Department of Genetics, University of North Carolina at Chapel Hill, Chapel Hill, North Carolina, United States of America; 2 The Jackson Laboratory, Bar Harbor, Maine, United States of America; 3 Lineberger Comprehensive Cancer Center, University of North Carolina at Chapel Hill, Chapel Hill, North Carolina, United States of America; University Hospital of the Canton Vaud (CHUV), SWITZERLAND

## Abstract

Genetic studies often seek to establish a causal chain of events originating from genetic variation through to molecular and clinical phenotypes. When multiple phenotypes share a common genetic association, one phenotype may act as an intermediate for the genetic effects on the other. Alternatively, the phenotypes may be causally unrelated but share genetic loci. Mediation analysis represents a class of causal inference approaches used to determine which of these scenarios is most plausible. We have developed a general approach to mediation analysis based on Bayesian model selection and have implemented it in an R package, bmediatR. Bayesian model selection provides a flexible framework that can be tailored to different analyses. Our approach can incorporate prior information about the likelihood of models and the strength of causal effects. It can also accommodate multiple genetic variants or multi-state haplotypes. Our approach reports posterior probabilities that can be useful in interpreting uncertainty among competing models. We compared bmediatR with other popular methods, including the Sobel test, Mendelian randomization, and Bayesian network analysis using simulated data. We found that bmediatR performed as well or better than these alternatives in most scenarios. We applied bmediatR to proteome data from Diversity Outbred (DO) mice, a multi-parent population, and demonstrate the power of mediation with multi-state haplotypes. We also applied bmediatR to data from human cell lines to identify transcripts that are mediated through or are expressed independently from local chromatin accessibility. We demonstrate that Bayesian model selection provides a powerful and versatile approach to identify causal relationships in genetic studies using model organism or human data.

## Author summary

Mediation analysis is a powerful tool for the discovery of causal relationships. Causal inference is increasingly being applied to genetic and genomic data to understand the chain of intermediate steps that propagate the effects of genetic variation. We describe a Bayesian model selection approach to mediation analysis, implemented in our bmediatR software. Using simulations, we show that bmediatR performs as well or better than established causal inference methods including the Sobel test, Mendelian randomization, and Bayesian network analysis. Bayesian model selection can incorporate prior information, provides greater flexibility in model specification, and can be tailored to address different inference questions. The model selection perspective helps to delineate the assumptions of causal analyses using the Sobel test or Mendelian randomization. We applied bmediatR to data from Diversity Outbred mice and human cell lines to demonstrate its ability to derive biologically meaningful findings in a variety of contexts.

## Introduction

Mediation analysis seeks to understand a causal process by determining whether an intermediate variable (M) explains (at least partially) the response of a dependent variable (Y) to changes in an independent variable (X). Though the causal interpretations of mediation analysis are subject to a number of assumptions [1], it has been widely used in both the social and natural sciences [2]. In a biomedical context, mediation analysis has been used to investigate how gene expression mediates the effects of genetic variants on complex phenotypes and disease [3, 4]. It has been used to infer causal relationships between biomolecular phenotypes, such as transcripts, chromatin states, and proteins [5, 6]. Here we focus on mediating genetic associations on biomolecular phenotypes, including gene expression, protein abundance, and chromatin accessibility, in model organisms and human cell lines, but the mediation approach we introduce is broadly applicable.

Mediation analysis requires that X, M, and Y are measured in the same individuals, and its causal interpretation relies on assumptions that are not verifiable based solely on the data [1, 7, 8]. In particular, it assumes that the direction of causal effects is from X to Y (X→Y), referred to as the direct effect of X on Y, and from X to M to Y (X→M→Y), referred to as the indirect effect of X on Y. It also assumes that these relationships are not subject to unobserved confounding, that observed confounders are conditioned on, and that observed confounders of M to Y do not depend on X. If these assumptions are satisfied, evidence for causal mediation lies in the magnitude of the indirect effect. If this indirect effect is non-zero, then M is a mediator of X on Y. Further, if M is a mediator and the direct effect is zero, then M is a complete mediator of X on Y, whereas if the direct effect is non-zero, then M is a partial mediator of X on Y. Our objective is to assess evidence of complete or partial mediation including the case where X comprises more than one independent variable.

Our motivating context is quantitative trait locus (QTL) mapping, where a trait of interest Y (*e.g.*, protein abundance) is associated with genetic variation represented in a matrix X, which could encode multiple variants or multi-state haplotypes. In particular, we are interested in assessing whether one or more candidate variables M may mediate the relationship between the genetic matrix and the trait. For example, a candidate mediator M could be the protein abundance of a gene encoded nearby a QTL for Y. In this case, it is reasonable to assume that the direction of causal effects is X→M→Y, and we further assume that the there is no unexplained confounding of these relationships. With the assumptions of mediation satisfied, our objectives are two-fold: 1) assess the evidence in favor of M being a causal mediator, and 2) determine if the mediation is partial or complete, given that X contains complex genetic information.

Traditional methods for mediation analysis are poorly suited to the dual objectives of detecting mediation and distinguishing complete from partial mediation when there are many candidate mediators and when X is a matrix. A classic approach for establishing mediation was introduced by Baron and Kenny [9]. This approach, termed the causal steps (CS) method, establishes evidence for partial or complete mediation by sequentially testing the relationships between X, M, and Y. Specifically, CS uses linear regression models to establish the following four conditions: 1) X has a marginal effect on Y [X→Y]; 2) X has an effect on M [X→M]; 3) M is at least a partial mediator of the effect of X on Y [M→Y|X]; and 4) M is a complete mediator of the effect of X on Y [X⫫Y|M]. The CS method can accommodate a matrix of independent variables by using a likelihood ratio test for grouped predictors. Although the CS method is useful due to its conceptual accessibility, its implementation in a genomics setting with many candidate mediators can be awkward. In particular, it is not straightforward to combine statistics across the steps while also accounting for multiple testing, particularly for step (4), which requires a failure to reject a null hypothesis. This makes it difficult to succinctly summarize evidence for complete or partial mediation for many candidate M.

Other common tests for mediation analysis address the problems of the CS method by providing a single test statistic for the significance of the indirect effect. The indirect effect is formally given as the product of regression coefficients from X → M and M → Y | X, that is, the effect of M on Y controlling for the effect of X. Establishing that this coefficient product is non-zero gives evidence for (at least) partial mediation, but it does not provide information about complete mediation. The most popular methods for testing the indirect effect include the Sobel test [10], which is based on an approximation to the asymptotic distribution of the indirect effect; alternatively, bootstrapping can be used to assess significance [11], which does not make distributional assumptions but is computationally expensive. In addition to not providing information about complete mediation, the Sobel test does not generalize when X is a matrix.

Applications of mediation analysis to large scale genetic and molecular profiling data have used modified versions of the traditional tests described above. Approximations to CS have been used in the multi-parent Collaborative Cross (CC [6, 12, 13]) and Diversity Outbred mouse populations (DO [5, 14–16]). These studies identified QTL for gene expression (eQTL), protein abundance (pQTL), and chromatin accessibility (cQTL). Detection of a significant QTL for the target phenotype (Y) satisfies step (1) and detection of a QTL local to the molecular trait (M), *i.e.*, near the genomic position of M, satisfies step (2). For a given phenotype QTL, a mediation scan is performed by testing the effect of X on Y as being mediated through each M (*e.g.*, each observed gene transcript). For the approximation to CS, significant mediators are determined based on the reduction in log-odds (LOD) score before and after accounting for the effects of M (hereafter referred to as LOD drop). This approximates step (4) without requiring complete independence between X and Y. Notably, the LOD drop method does not directly check step (3) and, as a result, it may detect candidates that are correlated with the true mediator but are not mediating the effect of X on Y. Thus, care is needed when interpreting the LOD drop mediation.

More recent methodological developments in large-scale mediation of genetic and molecular profiling data include the multi-SNP intersection union test [17], an extension of the CS method that simultaneously models the overall effect of multiple genetic predictors by representing them as a similarity matrix-based (*i.e.*, kernel-based) random effect, and the divide-aggregate composite null test [18], an extension of the joint significance test [19] that improves power relative to the Sobel and joint significance tests by utilizing an empirical null distribution. As with other methods based on the indirect effect, neither of these methods provide inference on distinguishing partial and complete mediation.

Instrumental variable (IV) analysis is a closely related but distinct approach to causal inference that, while dependent on its own strong assumptions, is more robust to the presence of confounding variables than mediation. Commonly referred to as Mendelian Randomization (MR) [20, 21] in genetic studies, it tests for a causal effect from M to Y using the inferred causal effects of instrumental variables—in this case genetic variants—on M and Y. MR methods remain an active area of development [22–26]. Importantly, it assumes there is no direct effect of X on Y, *i.e.*, that all intermediates are complete mediators; this strong assumption allows MR to avoid the potential pitfall of CS step (3) (M→Y|X), which can produce false mediators in the presence of confounding variables. Nonetheless, although robust to confounding, the assumption of no direct effect of X on Y may make MR poorly suited to some applications, including analyses motivated by an initial detection of a marginal association between X and Y.

All of the approaches described above rely on hypothesis testing, in which significance criteria are used to choose between nested alternative models. In our view, a more natural perspective for mediation analysis is provided by Bayesian model selection. Specifically, the goal of mediation analysis is to classify the relationship between X, M, and Y as a particular causal model. We note that this involves overcoming several challenges: the set of potential causal models is not nested; the classification into a particular causal model with finite data is necessarily uncertain; and estimation of parameters when the model is uncertain ideally requires incorporation of model uncertainty into the estimate. These challenges are coherently addressed by the Bayesian model selection paradigm, which considers a set of potential models (nested or otherwise) and assigns a posterior probability to each model.

Bayesian methods have been used in mediation analysis to estimate the posterior distribution of the indirect effect [27–29], and Bayesian model selection has been used to test for the presence of an indirect effect [30]. Here we develop a Bayesian model selection approach that considers a user-specified set of causal models, and that is capable of distinguishing between complete mediation, partial mediation, and independent effects of X and M on Y. The latter is particularly important in genetic studies where chance co-localization of genetic effects can be misinterpreted as causal associations. Mediation in this context can be seen as a small scale, focused version of causal network analysis (also known as Bayesian networks [31]) with genetic anchors [32–34], and Bayesian methods for inferring such networks are typically highly computationally intensive [35, 36]. By focusing on mediation and employing conjugate priors, we avoid costly sampling techniques for calculating posterior summaries. Our approach is computationally efficient, and it provides informative summaries of posterior model probabilities.

## Results

**Fig 1:**
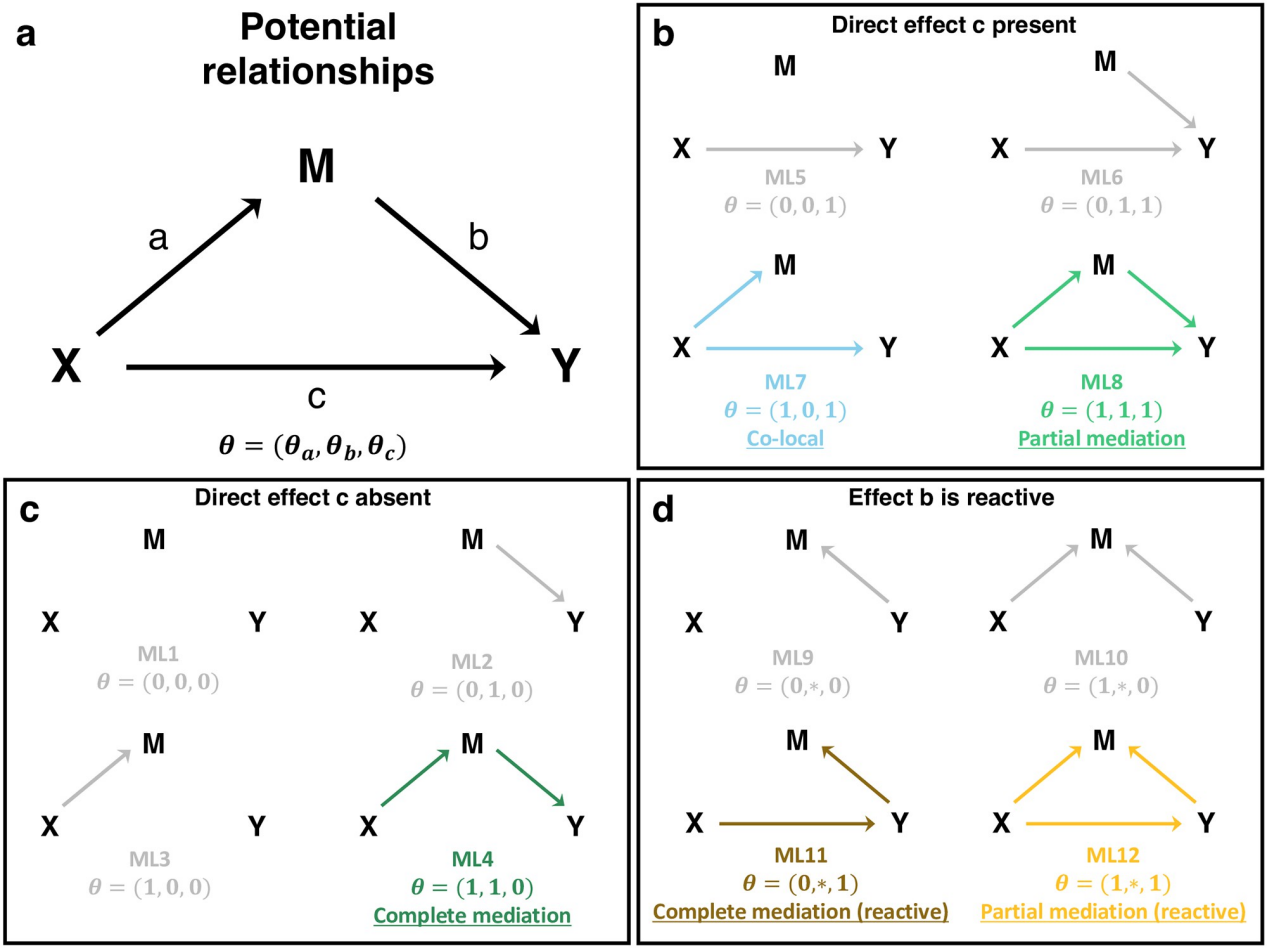
Possible relationships among X, M, and Y. X is assumed to be exogenous, and thus M and Y have no effects on X. A model and corresponding marginal likelihood (ML) are defined by the presence or absence of any of the three edges *a*, *b*, and *c* according to an indicator variable ***θ***. In this work and by default in bmediatR, the direction of edge *b* is assumed to be from M to Y (M → Y), but a set of reactive models can also be accommodated in which the direction of edge *b* is reversed (M ← Y), indicated with ***θ*** = (*θ*_*a*_, *, *θ*_*c*_). Models can be favored or even excluded by adjusting the model priors. By default, there are five models (ML1–3 and ML5–6) that represent non-mediation, *i.e.*, the effect of X on Y, if present, is not mediated through M. The co-local model (ML7) represents a special case where there is no mediation between X and Y, but X independently affects M and Y. The complete mediation model (ML4) and the partial mediation model (ML8) represent cases where the effect of X on Y is explained, completely or partially, by the effect of X on M.

We developed a Bayesian model selection approach, implemented in the R package bmediatR, to evaluate alternative causal models that define relationships between a continuous dependent variable Y, an independent (or exogenous) variable X, and a continuous mediator variable M. The causal models can be described as directed acyclic graphs (DAGs) (Fig 1). There are three possible edges (*a*, *b*, *c*) and we define each to be either present or absent using an indicator vector ***θ*** = (*θ*_*a*_, *θ*_*b*_, *θ*_*c*_), where for example ***θ*** = (1, 0, 0) denotes presence of *a* only. This leads to eight possible combinations of edges, each defining a different causal relationship (causal model) (ML1–8 in Fig 1). As an extension, the Bayesian model selection approach can also consider four additional causal models in which the direction of edge *b* (between M and Y) is reversed (ML9–12 in Fig 1, termed “reactive” models), though some of these are not causally identifiable (see Methods).

We are primarily interested in models that describe mediation, defined as any causal relationship where edges *a* and *b* are both present, *i.e.*, ***θ*** = (1, 1, *θ*_*c*_), indicating a causal path from X to Y through M. Complete mediation describes models where X acts entirely through M and thus X conveys no additional information about Y beyond that provided by M (*θ*_*c*_ = 0); partial mediation describes models where X conveys additional information beyond that contained in M (*θ*_*c*_ = 1); and there is no mediation when *θ*_*a*_ = 0 or *θ*_*b*_ = 0, which includes the co-local model in which X affects both Y and M but independently, and also includes the model in which X and M affect Y but are independent of each other. Our approach calculates a posterior probability for each causal model ***θ*** given data on X, M, and Y.

Bayesian model selection requires specifying prior probabilities for the models under comparison (model priors), as well as prior distributions for the parameters of those models (effect priors). Priors can be used to incorporate external information into mediation analysis, which can improve power and reduce false positive rates. Since the results of Bayesian model selection analysis can be sensitive to the choice of priors, it is important to understand the role and interpretation of different prior specifications. The model priors are specified by assigning probabilities to each of the possible configurations of ***θ*** (Fig 1). These probabilities can be viewed as weights and modified to define informative prior expectations on the relative frequency of different causal models. Setting a model prior to zero removes it from consideration, and this allows a user to define a set of allowable models. By default, we assign equal prior probability to the models that assume no reverse causality from Y to M for edge *b* (*i.e.*, probability 1/8 for each model ML1–8 but probability zero for ML9–12).

Conjugate prior distributions on the effect sizes of the edges connecting X, M, and Y (effect priors) yield a closed form for the joint likelihood of M and Y. The size of the effects is controlled by specifying the hyperparameters ***ϕ***, which are ratios of the edge effect sizes to the variability of the errors (see Methods). The default specification ***ϕ*** = (1, 1, 1) assumes that all edges have *a priori* equal effect sizes, also equal in size to the error variances of M and Y. Conjugate priors and specifying the edge effect sizes make computing the posterior fast and exact.

### Bayesian model selection performance in simulated data

Using simulated data, we evaluated our Bayesian model selection against four alternative causal inference methods: the Sobel test, LOD drop, IV analysis (ivreg [37]), and Bayesian network analysis (bnlearn [38]). Data were simulated by first specifying X and then simulating M and Y according to linear models with normal error and effect sizes, expressed as proportion of variation explained, that ranged from 0.05 to 0.95. This approach allows X to represent a non-normal (and non-scalar) quantity. In particular, for genetics applications, X may represent minor allele counts, multi-state founder haplotype probabilities, or multiple genetic variants. Data were simulated for five scenarios: co-local, where X drives M and Y independently; partial mediation, where X drives Y both directly and indirectly through M; complete mediation, where X drives Y only through its effect on M; and reactive versions of partial and complete mediation, where X drives M through Y instead. For each scenario, we simulated 100 data sets with 200 observations each. For each simulated data set, we applied bmediatR’s default model priors (*i.e.*, uniform over ML1–8 in Fig 1). We also considered two other types of model priors: reduced model priors, which sets a uniform prior probability over models that assume the direct effect *c* (ML4–8), and which is analogous to testing the indirect effect like the Sobel test; and expanded model priors, which sets a uniform prior over all models (ML1–12), including reactive ones (ML9–12) (S1 and S2 Figs). Furthermore, we varied effect size priors, using the default setting of equal sizes (50%) as well as an empirical setting for the size of each edge (see Methods).

It should be noted that all these methods provide different inferences. The Sobel test does not distinguish between partial and complete mediation, or their reactive forms. IV analysis and LOD drop method do not distinguish between co-local, complete, partial mediation, or the reactive forms of the mediation models. The set of models that can be distinguished by Bayesian model selection and Bayesian network analysis will depend on which model priors are non-zero. If a direct effect from X to Y is assumed (edge *c* fixed), these methods no longer distinguish partial and complete mediation, similar to the Sobel test. Alternatively, it is possible to include reactive models as non-zero prior models in situations when the effect from Y to M is plausible, though inference between some models is not causally identifiable (see Discussion).

#### Bayesian model selection performs well when genotypes are correctly specified

**Fig 2:**
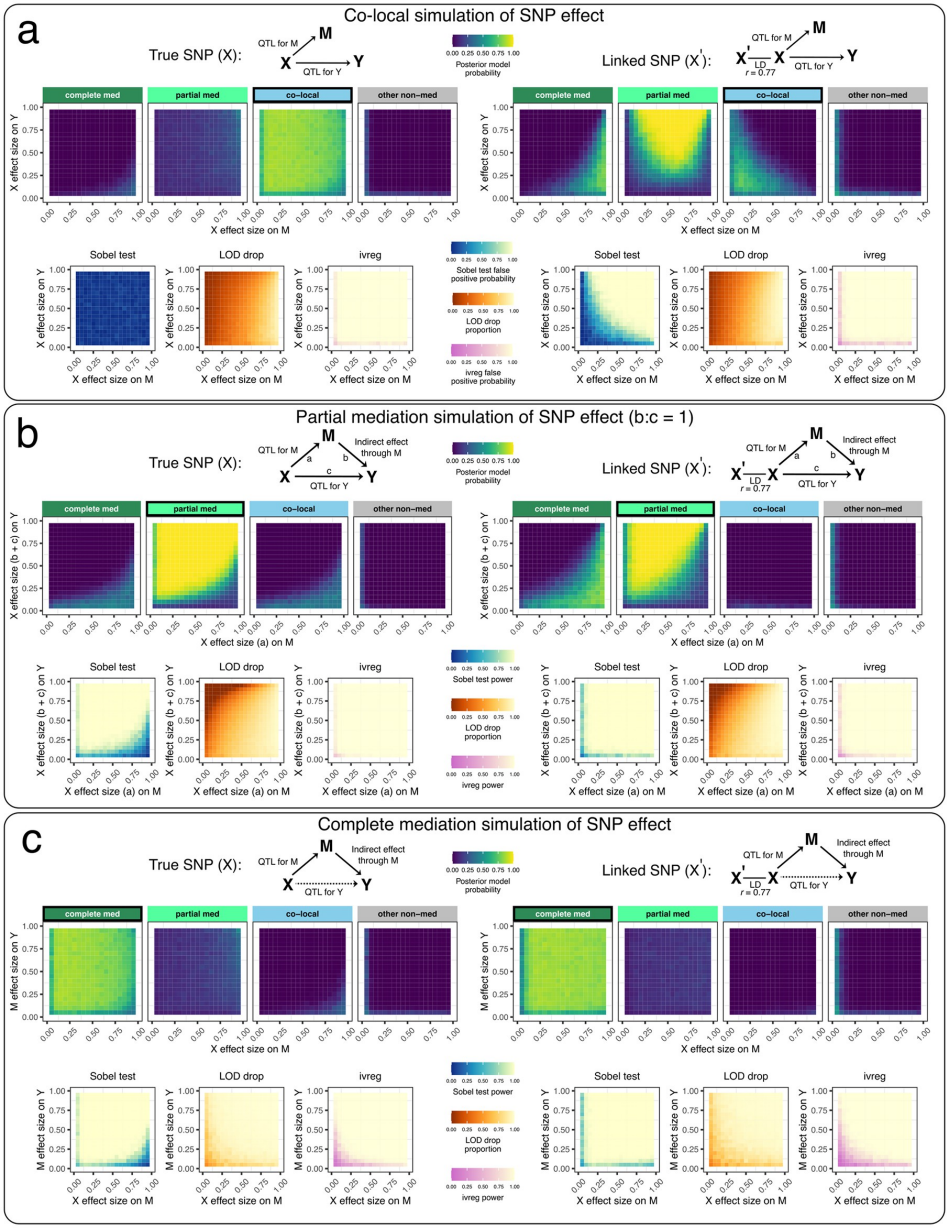
Performance of Bayesian model selection, Sobel test, LOD drop, and IV regression in simulated data with a binary exogenous variable. Data for 200 individuals were simulated according to (a) co-local, (b) partial mediation, and (c) complete mediation models based on a balanced bi-allelic variant X. We applied causal analysis with X as the true variant (left) and as a variant in linkage disequilibrium (*r* = 0.77) with the true variant (right). DAGs indicate the model used to simulate the data. Heat maps for Bayesian model selection represent the mean posterior probability associated with each inferred model for a range of fixed settings of the model parameters as indicated on x- and y-axes, each simulated 100 times. Heat maps for the Sobel test and IV regression represent false positive probability for co-local simulations and power for mediation simulations. Heat maps for LOD drop represent mean LOD drop, scaled to the proportion of the simulated QTL’s LOD score. See S1 and S2 Figs for Bayesian model selection results using empirical effect size priors and non-default model priors, including reactive. See S4 and S5 Figs for similar results from the other methods.

We evaluated the performance of the different mediation methods on simulated data with a bi-allelic QTL with an allele frequency of 0.5, for the co-local, partial mediation, and complete mediation models. Results from bmediatR and bnlearn with default priors are shown in Fig 2 and S3 Fig; results from all prior settings and all simulation settings, including reactive, are shown in S1, S2, S4 and S5 Figs. Note that although the summary statistics across methods are not the same and thus not directly comparable, they do represent the quantities researchers will use to draw inference in practice. Later, the methods are compared more directly using true positive and false positive rates.

The left panel of Fig 2A shows results when the SNP affects both M and Y but independently, as would be the case for an eQTL and a pQTL that are co-local but otherwise unrelated. The top four boxes show the posterior probability given by bmediatR for four types of models: complete mediation (ML4), partial mediation (ML8), co-local (ML7), and other non-mediation models (ML1–3 and ML5–6). Each box is a heat map of the posterior probability under all possible simulated effect sizes of X→M (x-axis) and X→Y (y-axis). Together, the four boxes show that, for all but the most extreme combinations of effect sizes, bmediatR overwhelmingly favors the (correct) co-local model. Below these are heat maps for the Sobel test, LOD drop, and IV analysis. The Sobel test and IV analysis aim to identify mediation, and so any detections for this co-local scenario represent false positives. The heat map show that false positive rates are low in all cases for Sobel test, but high for IV analysis. The LOD drop heat map shows the magnitude of the signal for mediation by that method. LOD drop correctly registers a minimal mediation signal when the effect size of the QTL for Y is large and that for M is small; but it incorrectly detects mediation when the QTL for M is large (>80%), a situation that is common in eQTL data.

The left panels of Fig 2B and 2C show results for the same set up but where mediation is present, either partial or complete. For partial (Fig 2B), bmediatR correctly identifies the type of mediation in most cases except for when the effect size of the QTL for M is disproportionately large, in which case it is misclassified as complete or co-local. The Sobel test correctly identifies the presence of mediation (type unspecified) with a similar error pattern as for bmediatR. LOD drop also performs well, albeit with different error characteristics. IV analysis performs the best at detecting partial mediation. For complete mediation (Fig 2C), the Sobel test and LOD drop perform slightly better than for partial mediation, IV analysis performs slightly worse than for partial mediation, and bmediatR accurately identifies the mediation type across all but the most extreme effect size settings. Bayesian network analysis performs almost identically to bmediatR when averaging across simulations (S3 and S6 Figs), which is unsurprising given they explore the same DAG likelihoods.

#### Misspecification of bi-allelic genotypes induces false mediation

Next we evaluated the mediation methods when the bi-allelic X is misspecified. This can occur, for example, when X represents a variant that is imperfectly correlated with the true causal variant through linkage disequilibrium (LD) (Fig 2, right panels). In this setting, Bayesian model selection favors the partial mediation model. Similarly, the Sobel test begins to detect mediation when it is not present, and IV analysis continues to incorrectly detect a causal effect in all settings. LOD drop also suffers with misspecification, exacerbating its issues when the QTL on M is large. When data were simulated with mediation present (partial and complete), misspecification of X was less problematic (Fig 2B and 2C right), though Bayesian model selection was less accurate at distinguishing partial and complete mediation.

#### Bayesian model selection and the Sobel test distinguish complete mediation from co-local and non-mediation

**Fig 3:**
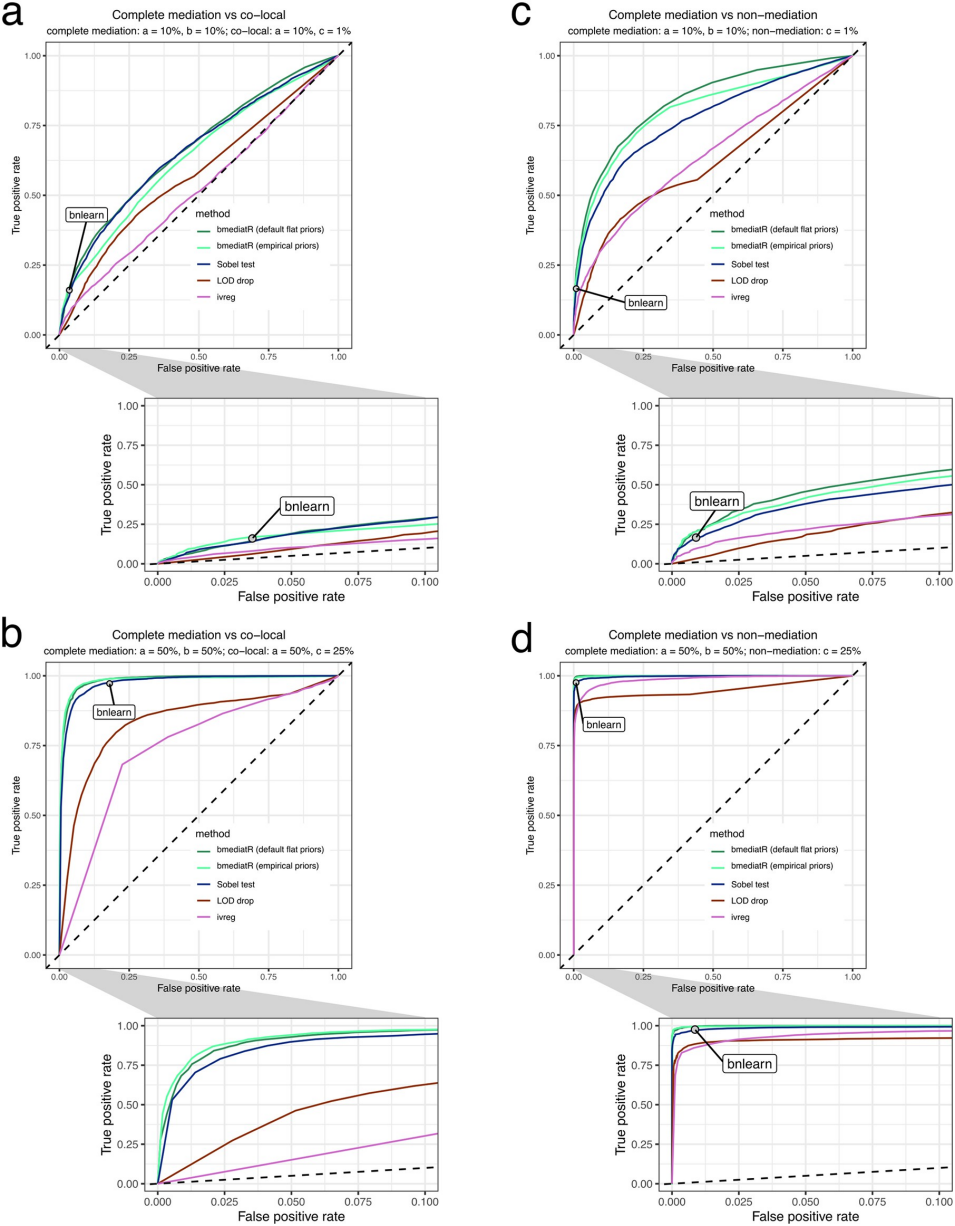
Performance of Bayesian model selection compared with other methods in distinguishing complete mediation from (a-b) co-local and (c-d) non-mediation. True positive rates (power) and false positive rates over a range of (*p*-value or posterior probability) thresholds were estimated from 5,000 simulations of 24 individuals according to a balanced bi-allelic variant X. Results are shown for data simulated with both (a-c) small genetic effects (X → M: 10%, M → Y: 10%) and (b-d) large genetic effects (X → M: 50%, M → Y: 50%). Diagonal dashed line is included for reference, representing a classifier with no ability to distinguish complete mediation from co-local or non-mediation. Note that bnlearn is represented by a point rather than a curve because (in our use of that method) it returns only a single, optimum model and so is not amenable to thresholding. See S7 Fig for methods’ performance in distinguishing partial mediation from co-local and non-mediation.

Given the differences in assumptions and output from these methods, we also summarized results based on true positive and false positive rates in the form of receiver operating characteristic (ROC) curves. Based on 5,000 simulations of 24 individuals, we compared the performances of bmediatR using both default and empirical effect size priors, bnlearn, the Sobel test, LOD drop, and ivreg in distinguishing complete mediation from co-local and non-mediation (Fig 3). Notably, the more formal mediation methods (bmediatR, bnlearn, and the Sobel test) performed the best, particularly at distinguishing complete mediation from co-local. The effect size priors did not strongly influence bmediatR’s performance. We performed a similar analysis comparing partial mediation to co-local and non-mediation (S7 Fig), which had similar results. See S1 Table for a description of the various methods evaluated and their features.

#### Misspecification of multi-allelic genotypes as bi-allelic induces false mediation

**Fig 4:**
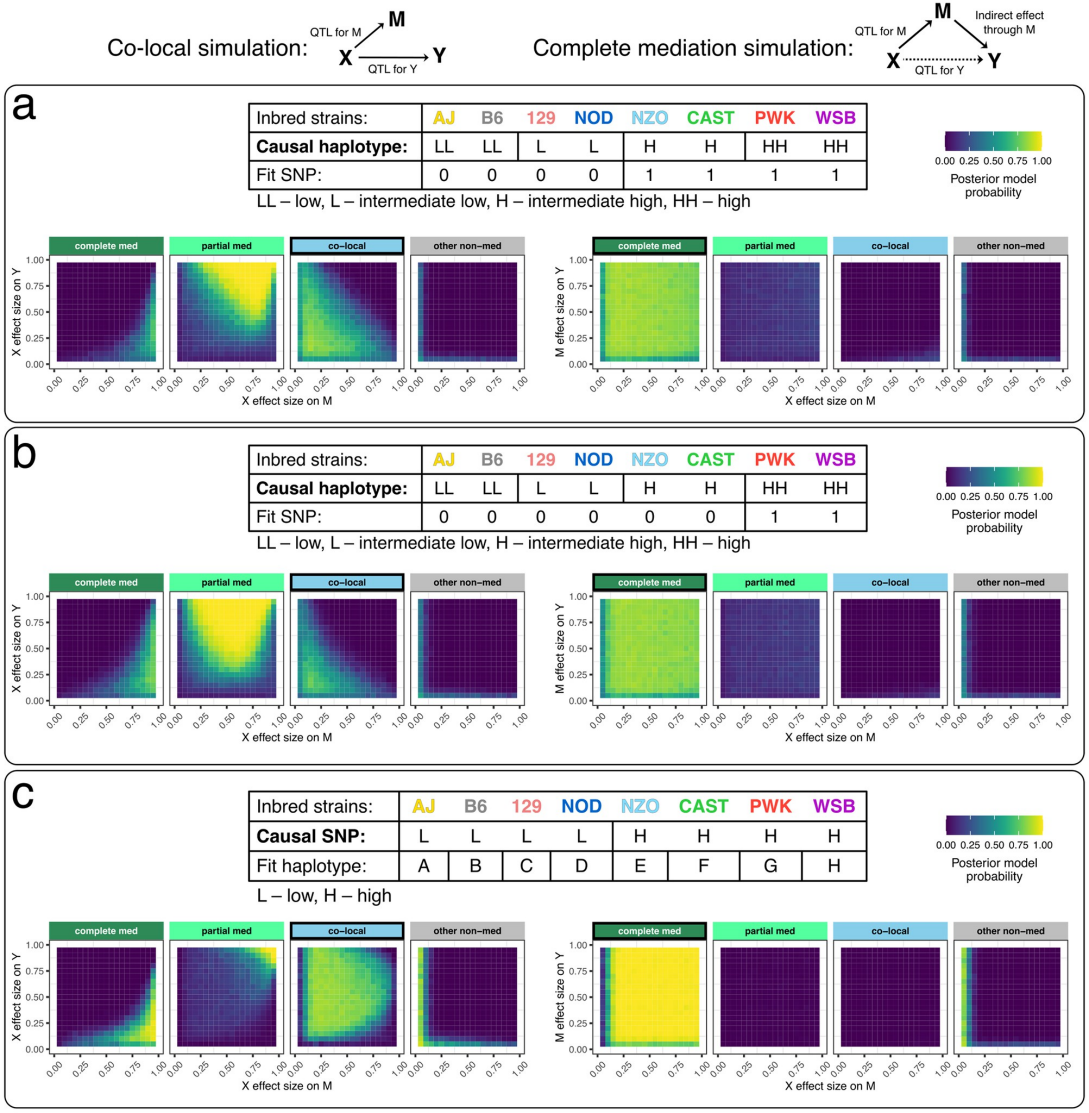
Performance of Bayesian model selection in simulated data with a multi-state exogenous variable. Data for 200 individuals were simulated according to co-local (left) and complete mediation (right) models. DAGs indicate the model used to simulate the data. (a-b) The genetic effect assumes four functional alleles with balanced allele frequencies (25%). Mediation analysis was performed using (a) a variant that tags the two higher functional alleles and (b) a variant that tags only the highest functional allele. (c) Data from a bi-allelic variant with allele frequency 50% were simulated, and mediation analysis performed using 8 founder haplotype states. The tables describe the structure of the genetic effect X used to simulate the data (causal) versus the X used in the mediation analysis (fit), in terms of the distribution of alleles among the founder strains. For example in (a), the low and intermediate low functional alleles are tagged by one allele of the fit SNP and the high and intermediate high functional alleles are tagged by the other allele of the fit SNP. Heat maps for Bayesian model selection represent the mean posterior probability associated with each inferred model for a range of fixed settings of the model parameters as indicated on x- and y-axes, each simulated 100 times.

Next we evaluated how mediation through Bayesian model selection is affected by misspecifying a multi-state haplotype effect. Data were simulated for 200 individuals with equal frequency of four alleles with distinct effects. Fitting X based on a bi-allelic SNP that tags the low and high haplotype groups worked well for complete mediation, but resulted in false mediation signals for co-local data, only correctly preferring the co-local model when at least one of the QTL on M and Y were small (<50%) (Fig 4A). This issue was exacerbated when the SNP was more imbalanced, resulting in false mediation signal for co-local data across a wider range of effect sizes (Fig 4B). We then looked at simulated bi-allelic data but modeled the genetic effect as eight haplotypes. Bayesian model selection performed well for co-local and complete mediation data, aside from some edge cases with extreme effect sizes, most notably at the corners of large QTL on M with small QTL on Y (prefers complete mediation) and small QTL on M and large QTL on Y (prefers partial mediation) (Fig 4C).

#### Bayesian model selection can detect mediation of multi-allelic QTL

**Fig 5:**
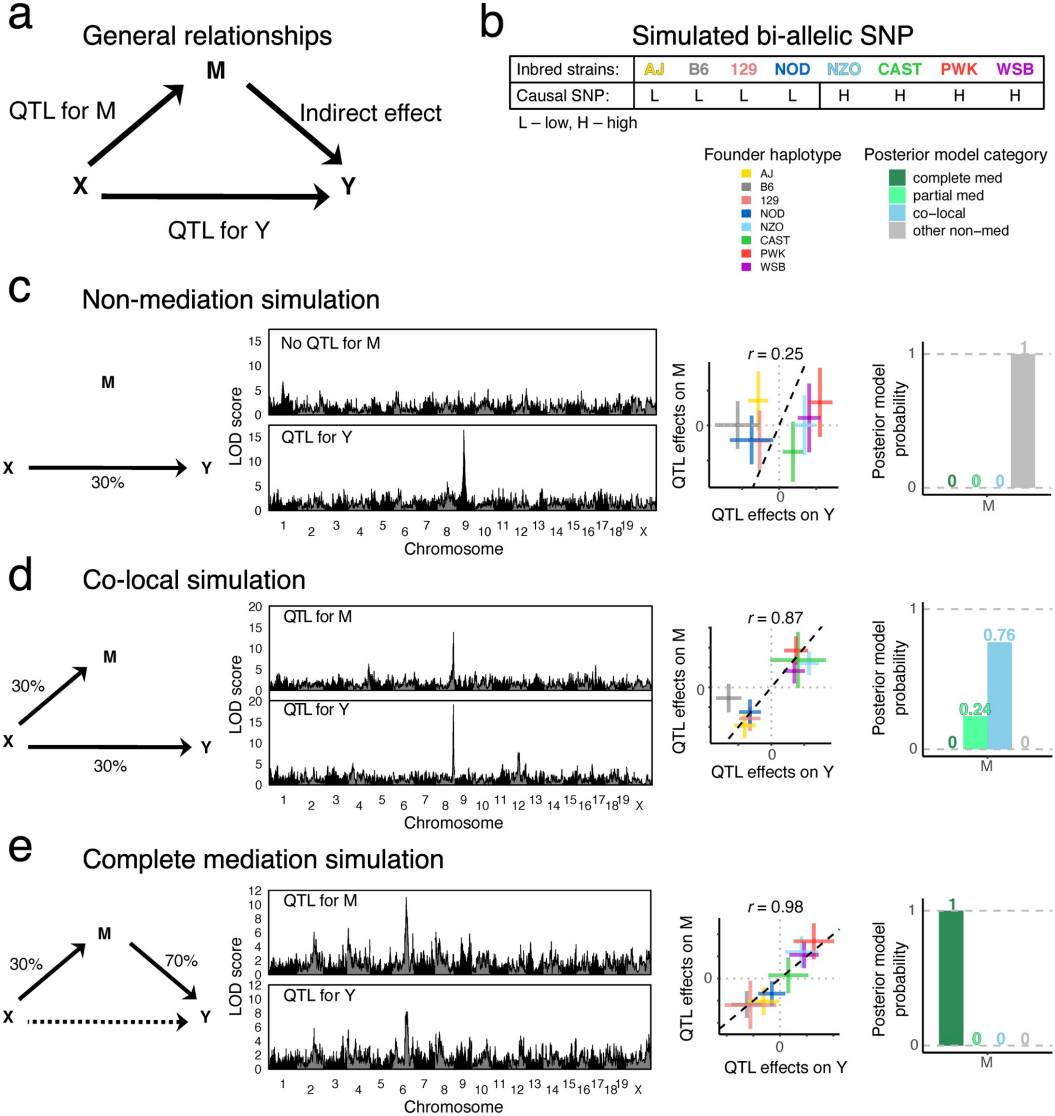
Illustration of Bayesian model selection applied to QTL mapping with simulated DO mouse data. (a) The DAG is labeled to indicate how each arm in the mediation model is interpreted in the QTL mapping setting. Y and M were simulated based on a bi-allelic QTL X at a randomly selected locus, with (b) each allele distributed to four founder strains. Genome-wide genotype data were obtained from 192 DO mice, according to one of three models: (c) M is a non-mediator of X on Y, (d) M and Y are independently driven by X (co-local), and (e) M is a complete mediator of X on Y, as illustrated with the corresponding model DAG with the simulated effect sizes indicated in units of percent variance explained (left). Genome-wide LOD scores for QTL mapping of M and Y, a scatter plot of the founder haplotype effects at the QTL for M and Y, and the Bayesian model selection posterior model probabilities are shown (from left to right).

To demonstrate mediation analysis in the context of multi-allelic QTL analysis, we simulated data based on the genomes of 192 DO mice [5]. A genetic locus X was randomly selected and M and Y were simulated assuming no mediation (QTL for Y, no QTL for M, M and Y uncorrelated; ML5), co-local (ML7), and complete mediation (ML4). QTL mapping was then performed for both M and Y to obtain LOD scores and estimated haplotype effects (Fig 5). The simulations demonstrate characteristic features of co-mapping QTL with correlated effects for co-local and complete mediation simulations, as they would appear in a multi-allelic QTL analysis. Data were simulated from a bi-allelic genetic variant, but the QTL mapping and Bayesian model selection were performed based on 8-state founder haplotypes.

### Bayesian model selection applied to DO mice

In order to illustrate how bmediatR works in QTL mapping applications, we analyzed previously reported liver proteomics data from 192 DO mice [5, 13]. We first illustrate how multi-state haplotypes help to identify the causal driver of a distal pQTL for *Snx4*. Then we look at a case in which bmediatR identifies a biologically plausible mediator for a distal pQTL of *Tubg1* where LOD drop favored a less plausible candidate.

#### Multi-state haplotypes improve mediation inference for SNX4 distal pQTL

**Fig 6:**
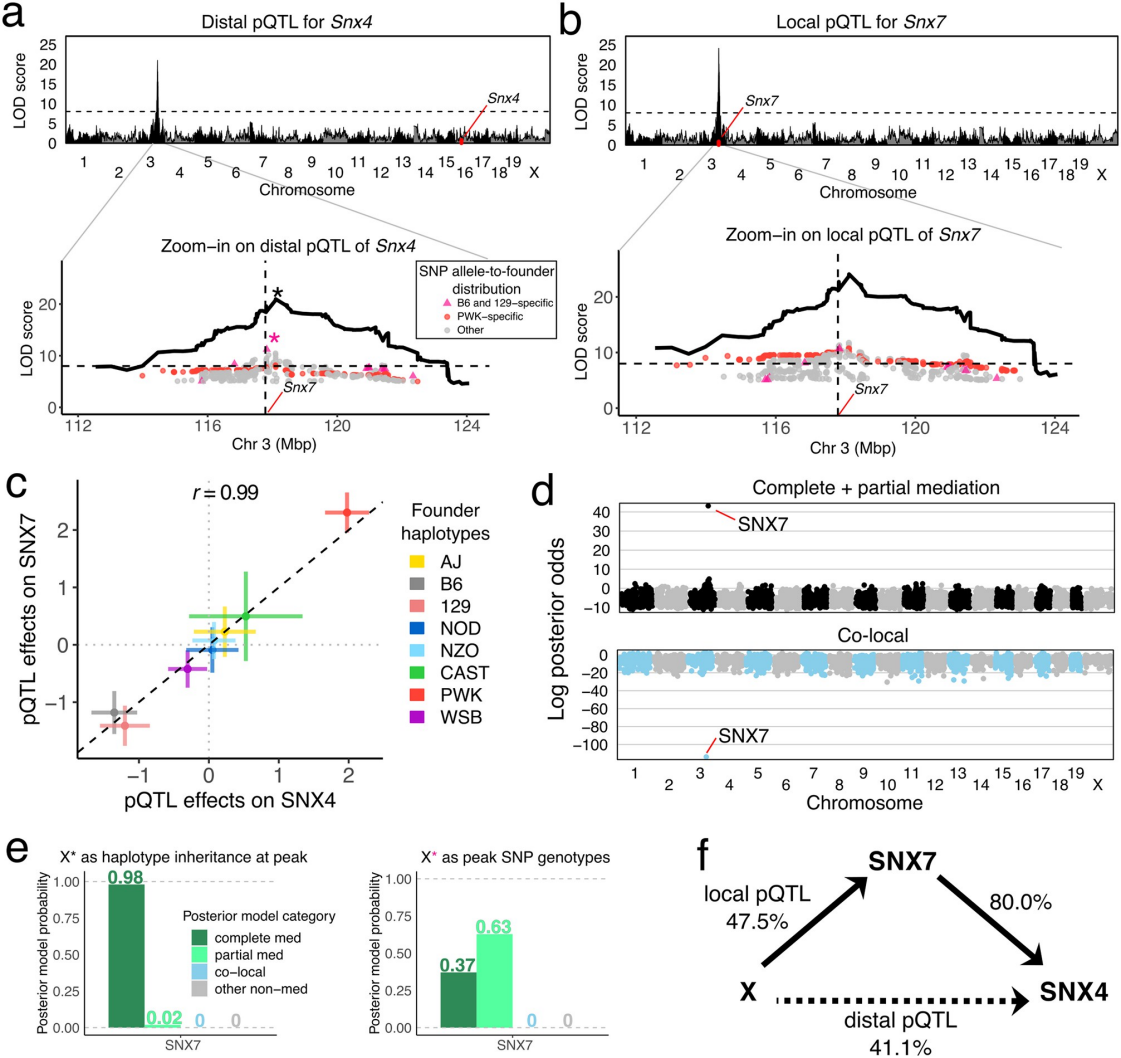
Mediation analysis of a distal pQTL for *Snx4* in DO mice. Genome-wide LOD scores for associations of (a) SNX4 and (b) SNX7 abundance were performed using founder haploptye linkage mapping. Zooming into the QTL region, LOD scores for variant association within the pQTL region (peak ± 5 Mbp) for bi-allelic vartiants with LOD scores > 5 are overlaid on the haplotype association LOD curve. Variants with alleles specific to B6 and 129 (pink) and PWK (red) are highlighted. (c) The founder haplotype effects at the pQTL are multi-allelic and highly similar for the two proteins. (d) Genome-wide mediation scan where all observed proteins are individually evaluated as mediators of the *Snx4* distal pQTL highlights SNX7 as a mediator (complete and partial summed) and strongly indicates that the co-local model is unlikely. Each point represents the log posterior odds for a candidate mediator for the specified mediation model. (e) Posterior probabilities of mediation models for the pQTL (left) using founder haplotypes and (right) using the peak bi-allelic variant. (f) The complete mediation model with SNX7 as mediator of the *Snx4* distal pQTL is shown as a DAG with estimated effect sizes in units of percent variance explained. The dashed line indicates the strength of the distal pQTL that is not included in the model because it is completely mediated.

The *Snx4* gene is located on chromosome 16 and has a distal pQTL on chromosome 3 that co-maps with a local pQTL for *Snx7* (Fig 6). The proteins for both genes are sorting nexins that bind phospholipids, form protein-protein interactions, and play a role in membrane trafficking and protein sorting [39]. The haplotype effects of the *Snx7* local pQTL and the *Snx4* distal pQTL are highly correlated (*r* = 0.99) and reveal a complex haplotype effects pattern that cannot be explained by a single bi-allelic variant. We used the TIMBR software [40] to determine that the pQTL has as many as ≈5 distinct functional alleles (S8 Fig). Bi-allelic variants in the pQTL region with alleles shared by the B6 and 129 strains partially match the haplotype effects and thus have strong associations with both proteins. We then evaluated all proteins (genome-wide) as potential mediators of the *Snx4* distal pQTL, and only SNX7 is identified as a likely mediator based on the log posterior odds (Fig 6D), and notably, is unlikely to be co-locally regulated. We also looked more closely at the posterior probabilities for SNX7 as a mediator of the pQTL effect (encoded in terms of founder haplotypes), and compared with mediation of the pQTL encoded as the peak SNP (with B6 + 129 allele). We found that the specific variant-based analysis assigns most of the posterior probability to the partial mediation model, whereas using the 8-state haplotype information finds strong support for complete mediation.

#### Bayesian model selection favors a biologically plausible driver for TUBG1

**Fig 7:**
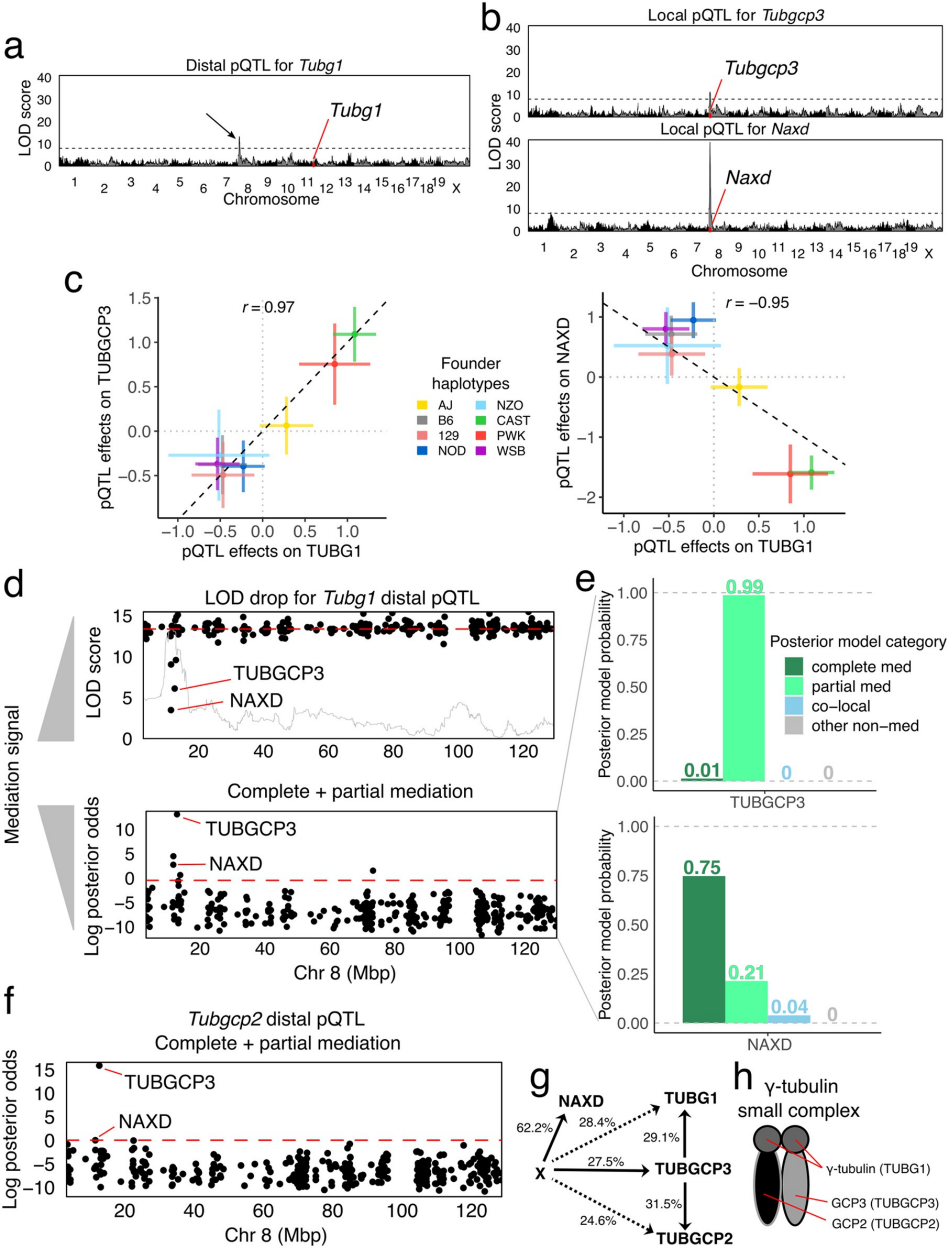
Mediation analysis of a distal pQTL for *Tubg1* in DO mice. (a) Genome-wide LOD scores for TUBG1 abundance. Black arrow indicates distal pQTL on chromosome 8. (b) Genome-wide LOD scores for two genes, *Tubgcp3* (top) and *Naxd* (bottom), with co-mapping local pQTL. (c) Comparison of the founder haplotype effects of the *Tubg1* pQTL with *Tubgcp3* (left) and *Naxd* (right) pQTL. (d) Mediation scans of all observed proteins on chromosome 8 by LOD drop with an overlay of the pQTL LOD scores in gray (top) and Bayesian model selection log posterior odds for mediation (bottom) show different prioritization for candidate mediators NAXD and TUBCP3. Note that low LOD drop scores indicate stronger mediation signal. (e) Posterior model probabilities for the *Tubg1* distal pQTL for candidate mediators (top) TUBGCP3 and (bottom) NAXD. (f) Mediation scan for the *Tubgcp2* distal pQTL identifies TUBGCP3 as the best candidate mediator. (g) The DAG summarizes the mediation analysis results with effect size estimates shown as percent variance explained. Dashed lines indicate the strength of distal pQTL effects that are not part of the model assuming complete mediation through TUBGCP3. (h) TUBG1, TUBGCP2, and TUBGCP3 comprise the *γ*-tubulin small complex.

The gene *Tubg1* is located on chromosome 11 and has a distal pQTL on chromosome 8. In our previous work, genome-wide mediation analysis by the LOD drop method identified NAXD as the best candidate mediator, but also revealed a significant LOD drop score for TUBGCP3 (Fig 7). Both mediation candidates have local pQTL. The *Naxd* local pQTL is stronger (LOD score ≈40) than both the *Tubg1* distal and *Tubgcp3* local pQTL (LOD scores ≈10). The pQTL haplotype effects for *Tubgcp3* and *Naxd* are highly correlated with the distal pQTL effects on TUBG1 (although flipped for NAXD) (Fig 7C). The allelic series could be either bi- or tri-allelic (*k* of 2–3; S8 Fig). We applied Bayesian model selection to both candidate mediators using the haplotype effects. The posterior probabilities at the two candidate mediators show that (Fig 7E) TUBGCP3 has 99% probability as a partial mediator, with the remaining probability for complete mediator. In contrast, NAXD, which is the best candidate using the LOD drop method, has 75% probability of being a complete mediator, 21% partial mediator, and 4% co-local probability. Due to the non-zero co-local probability of NAXD, TUBGCP3 is preferred based on the combined posterior odds of partial and complete mediation. A related tubulin gene, *Tubgcp2*, encoded on chromosome 7, has a distal pQTL at this locus on chromosome 8 (Fig 7G). Bayesian model selection analysis of the *Tubgcp2* distal pQTL also supports TUBGCP3 as a candidate mediator. These findings support a model for stoichiometric co-regulation of the protein constituents of the tubulin small complex [41, 42] that is mediated by the abundance of TUBGCP3.

### Mediation of genetic effects on gene expression through chromatin accessibility in human cell lines

We applied Bayesian model selection to data from 63 human lymphoblastoid cell lines (LCLs) using genotype [43], RNA-seq [44], and DNase-seq [45, 46] data. We used both Bayesian model selection and the Sobel test to identify chromatin regions near transcription start sites that may act as mediators of gene expression. Previously, genetic variants that affect chromatin accessibility and gene expression were mapped in the LCLs [45]. Variants associated with chromatin accessibility were also likely to be associated with the expression of nearby genes, indicating that chromatin accessibility may be a common mechanism by which transcription is regulated.

**Fig 8:**
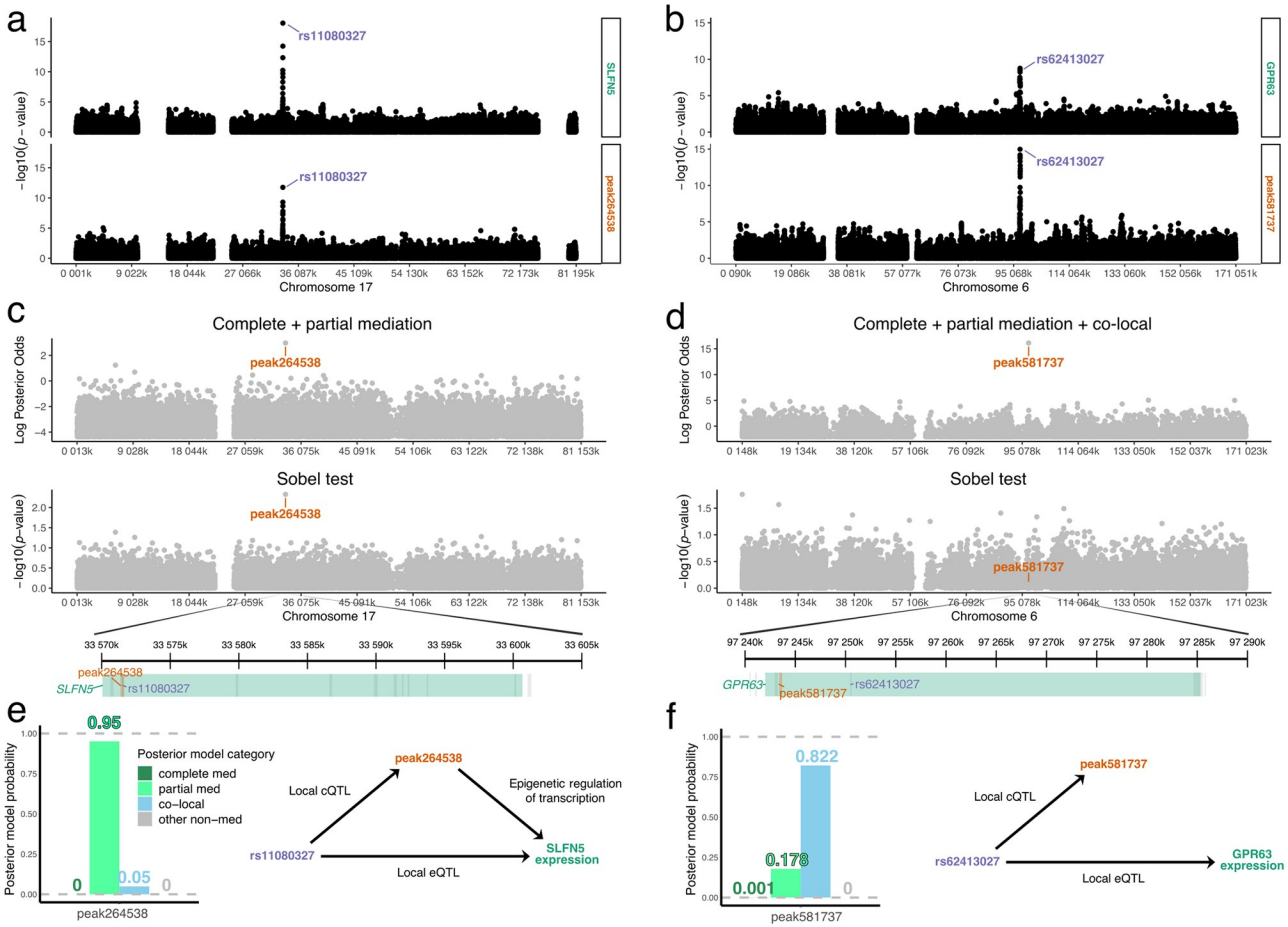
Mediation analysis of local chromatin state and gene expression data in human cell lines. SNP associations with (a) SLFN5 and (b) GPR63 expression (top) and nearby chromatin accessibility (bottom) for variants on the genes’ chromosome. Peak SNPs are labeled. Mediation results for the (c) SLFN5 eQTL and (d) GPR63 eQTL. Log posterior odds from Bayesian model selection (top), -log10 *p*-values from Sobel test (middle), and zoomed-in window highlighting gene start, peak SNP, and peak mediator (bottom). Peak mediator or co-local chromatin peak is labeled. Each gray point represents a chromatin peak candidate mediator located near the gene of interest. For SLFN5 expression, complete and partial mediation models were summed in the posterior summary from Bayesian model selection. For GPR63 expression, the co-local model was also summed with the mediation models. Posterior model probabilities from Bayesian model selection for the peak mediator and co-local chromatin peaks and the implied DAG for the (e) SLFN5 eQTL and (f) GPR63 eQTL.

To illustrate, we looked at the expression of gene SLFN5 which was strongly associated with local genetic variation (eQTL). These genetic variants were also strongly associated with chromatin accessibility (cQTL) (Fig 8A). The SNP most strongly associated with SLFN5 expression and chromatin accessibility is located within an interferon-stimulated response element (ISRE) in the first intron of SLFN5 [45], and the co-mapping chromatin site is directly above the SNP. The juxtaposition of chromatin site to gene makes it a likely mediator for the expression of SLFN5, an interferon-regulated gene, because it controls the accessibility of the ISRE to transcription factors [47]. Bayesian model selection and the Sobel test both detect partial mediation.

Co-mapping eQTL and cQTL can also represent independent signals. A SNP within 10 Kbp of the start of GPR63 is a local eQTL and a local cQTL for a nearby chromatin site (Fig 8B). The Sobel test does not support the chromatin site as a mediator of the genetic effect on GPR63 expression. Bayesian model selection results are consistent with the Sobel test in ruling out mediation but, unlike the Sobel test, it clearly identifies the relationship as co-local.

## Discussion

Results from our Bayesian model selection analysis can be sensitive to the choice of model prior; therefore, it is important to understand the role and interpretation of different prior specifications. In our framework, it is possible to define any configuration of prior weights over a set of models using the model prior. For example, if one believes that X is not a QTL for most candidate mediators, we could assign a smaller prior probability to models that include the *a* edge; or, if we believe that reactive models are unlikely but do not want to exclude them completely, we could assign these models a small prior probability. The appropriateness of such choices depends on the context of the analysis and the set of candidate mediators under consideration, for example, if we are considering only genes nearby X as candidate mediators, versus all genes in the genome.

A straightforward elaboration of our approach is to use a set of candidate mediators to learn an empirical prior distribution over the causal models. If suitably constructed, an empirical prior could improve power and reduce false positives within a given dataset. An empirical prior could be implemented, for example, by computing the model posterior for all candidate mediators using the uniform model prior, and then taking the average over all posteriors as the empirical prior distribution for downstream analyses. Conceptually, this approach is similar to the empirical null employed in Liu *et al* [18]. As an illustration, in the case where X is a QTL for Y, if most of the candidates are not actually mediators, an empirical prior would put a high prior probability on models that include the *c* edge and do not represent mediation. Our simulations indicate that the default uniform model prior led to reasonable model selection inference over a range of true effect sizes and causal models. That said, exploring alternative prior distributions over the causal models is an intriguing avenue for future research, and many strategies could be implemented within our flexible framework.

There may also be opportunities to improve inference by adjusting the effect size hyperparameters, ***ϕ***. By default, we assume *a priori* equal effect sizes for each edge, which are also equal to the size of the error variances. We also evaluated setting the effect size hyperparameters empirically, though this did not strongly influence performance. Tuning these hyperparameters to reflect prior beliefs about relative effect sizes may improve posterior model inference, provided these prior beliefs are closer to the truth. It is possible to put prior distributions on the effect size hyperparameters, but doing so would complicate posterior inference, as conditioning on these hyperparameters makes inference fast and exact. Another possibility for setting the effect size hyperparameters would be maximum *a posteriori* estimation (*e.g.*, via grid search).

Mediation analysis critically assumes that there is no confounding between M and Y, *i.e.*, M ← U → Y. In the presence of confounding, M and Y may be associated even if there is no direct effect from M to Y. Observed confounders can be adjusted for as covariates (**Z** in the Methods). Unobserved confounders present a more fundamental problem. Approaches that account for unobserved confounders [48] could be implemented, but only as part of a two-stage approach, where latent factors are inferred first, naive to the mediation model, and then either removed or included as covariates in the mediation analysis. In our real data applications, we analyzed experimental data from mice and human cell lines, which we assumed was unconfounded after controlling for sex, batch, and other experimental variables.

One alternative to mediation analysis that is robust to confounding is IV analysis, which is more commonly referred to as MR in genetics and genomics [20–23]. We evaluated MR as an alternative to our bmediatR mediation approach. MR involves regressing Y on an unconfounded version of M, specifically the predicted value of M estimated from X (X → M). Standard MR requires the strong assumption of no direct effect of X on Y (*i.e.*, no partial mediation), and as such it cannot be used to distinguish between partial and complete mediation. As demonstrated by our simulations, when the assumption of no direct effect is violated, MR struggles to distinguish mediation from co-localization (Figs 2 and 3, and S7 Fig), mistakenly inferring truly co-local relationships as causal. In our motivating context, mediation analysis is performed after detecting a marginal association between X and Y, and we are interested in evaluating many candidate M that may mediate this relationship. Given that X and Y are associated, we expect a direct effect from X to Y when evaluating “null” M that are not causally upstream of Y. Thus, the MR assumption of no direct effect will frequently be violated, making it poorly suited to such applications. Emerging MR methods relax the assumption of no direct effect and may be useful in this context [49–52]. This field is rapidly developing, and a fuller review of MR is beyond the scope of this work.

A different, non-causal method for assessing the relationship between M and Y is colocalization analysis [53–57], which evaluates if two variables (M and Y) share a common X, when only one of many independent variables are under consideration. This approach is typically motivated by fine-mapping genetic variants (*i.e.*, evaluating candidate X, rather than candidate M), and it does not establish a causal relationship between M and Y in the way that mediation analysis can.

In our simulations, Bayesian network analysis consistently identifies the model with maximum posterior probability from Bayesian model selection (S3 Fig). This suggests that bmediatR generalizes to a three node Bayesian network with full posterior inference. The value of bmediatR’s posterior inference is highlighted in S6 Fig, in which bmediatR reveals uncertainty in distinguishing causal models, whereas bnlearn, or at least our use of it, returns a single model solution without quantifying a corresponding level of confidence.

That said, our implementation of Bayesian network analysis is admittedly superficial: we use the bnlearn algorithm to find the best model, essentially inferring a point estimate in the model space, but more sophisticated applications of bnlearn attempt to quantify the frequentist variability of that estimate, and functions of it, through resampling. In particular, recent work by [32, 33] implements earlier recommendations of [31] to report an “averaged network”, created by averaging best models inferred from many boostrap resamples of the data; this procedure, a form of bagging [58], yields probabilities of inclusion and direction of each edge, which are then thresholded to yeild a final network estimate. The results from the resampling could also, in theory, be used to provide probabilities of any one particular model, providing a frequentist analog to our posterior model probabilities.

It is worth considering how these two types of model probabilities differ conceptually. The posterior model probabilities from bmediatR seek to quantify uncertainty about the true model arising from prior uncertainty specified by the researcher, whereas the resample-based probabilities seek to quantify the variability of an estimator, namely, a chosen best model, with respect to finite sampling, where finite sampling is approximated by bootstrapping (although other related methods have also considered subsampling [59–61] or variants of the Bayesian bootstrap [62]). A full comparison of Bayesian vs resample-based probabilities in this context is beyond the scope of this study. However, we do note a practical contrast that in the Bayesian approach the priors can be specified arbitrarily, whereas in the resample-based approach no such specification is available nor required.

A second way our approach differs from conventional Bayesian networks is the use of group-level (rather than variable-level) selection on X. For multi-allelic genotypes, such as those used in populations like the DO, this is an appealing feature because the removal of a column of X, representing a founder allele, would be difficult to interpret.

Mediation analysis commonly assumes that the direction of causal effect is from M to Y. This assumption must be justified by the context. For example, the mediator of a distal pQTL is expected to possess a corresponding local QTL. In the presence of reverse causality from Y to M, however, standard mediation analysis (excluding the reactive models) will still detect an edge from M to Y, yielding spurious evidence for mediation. In our framework, it is straightforward to include the reactive models in settings where reverse causality is a possibility. We examined inference in the presence of reverse causality via simulation (S1 and S2 Figs). These results demonstrate that it is possible to distinguish complete mediation from reactive complete mediation, but also indicate that it is not possible to distinguish partial mediation from reactive partial mediation. This is not unexpected, as these models are not identifiable. Our simulation results also show that, for the priors we specified, true partial mediation can appear more consistent with reactive complete mediation than partial mediation, for particular effect sizes (S1 Fig). These findings emphasize the importance of the assumption that M → Y in mediation analysis. By default, our bmediatR software excludes reactive models, and we recommend interpreting inferences made using the reactive models with caution.

Bayesian model selection provides an opportunity for more flexible model specifications than are possible for other methods, such as the Sobel test. In particular, the exogenous variable X can be categorical (with more than two groups) or multivariable. This is useful for modeling genetic effects in some contexts, as we demonstrated by the improvement in mediation using 8-state haplotypes in the DO mouse data. Mediation analysis with multiple exogenous variables [63] is easily implemented in bmediatR. The bmediatR software offers a framework that could be generalized to account for moderated mediation [9, 64, 65], in which the effect of the mediator is moderated by another factor. Increasing the complexity of the model space can increase computation time and may require more complex posterior summaries. Despite these hurdles, compared with methods like the Sobel test, implementing more general mediation model settings is straightforward within the Bayesian model selection framework.

Another possible extension of our mediation approach is to allow multiple mediator variables. In our current implementation of bmediatR, we only consider a single variable M that mediates the relationship between X and Y. It may be the case, however, that this relationship has multiple intermediates, making it desirable to evaluate evidence in favor of multiple mediators. Conceptually, it would be straightforward to extend our Bayesian model selection framework to include additional causal models that permit Y to depend on any combination of mediator variables. If the number of mediator variables under consideration were small (or if the model space were otherwise constrained, *e.g.*, consideration of only pairs of mediators), it would be possible to fully explore this extended model space and compute an analytic posterior distribution over combinations of mediator variables. If there were more than a few mediator variables under consideration, though, an exhaustive approach would be intractable, necessitating approximate posterior inference methods for variable selection such as through Gibbs sampling [66] or the more expansive network approaches of [35, 36]. Posterior inference via sampling would be a substantial deviation from the analytic posterior inference used in bmediatR and would introduce all the usual challenges of approximate posterior sampling, such as ensuring proper mixing of the parameters. Alternatively, our existing model could be used to evaluate evidence for multiple mediator variables by forward model selection. For example, if an initial mediation analysis yielded evidence in favor of a mediator variable, this mediator could be “selected” and included as a covariate for Y in a subsequent conditional analysis to evaluate an additional mediator. If the conditional analysis indicated support for another mediator variable, this would represent evidence in favor of multiple mediator variables. This procedure could be repeated, with multiple mediators selected as covariates for Y, until there was no evidence for additional mediator variables. This stepwise approach for identifying multiple mediator variables would be approximate, but could be implemented using our existing software. Nevertheless, we have not evaluated bmediatR in the context of multiple mediators as part of this study.

Lastly, we caution that mediation analysis is sensitive to misspecification. In our simulations, we showed that when a genotype X is misspecified (*i.e.*, a haplotype is incompletely described by a single variant), a true co-local model can be inferred as mediation. Simulations in other studies have demonstrated that greater precision in the measurement of Y relative to M can lead to true complete mediation appearing as partial mediation [67]. These issues highlight the need for careful examination and independent validation of inferences based solely on mediation analysis.

In conclusion, mediation analysis is a powerful tool for discovery and integrative analyses of high dimensional biological data, with the caveat that proper caution and awareness of the potential pitfalls are needed. Here we describe a flexible Bayesian model selection approach to mediation analysis that is implemented as the R package bmediatR. Using simulations, we show that bmediatR performs as well or better than established methods including the Sobel test, while allowing greater flexibility in both model specification and in the types of inference that are possible. We applied bmediatR to genetic data from mice and from human cell lines, demonstrating its ability to derive biologically meaningful findings. The Bayesian model selection approach provides a flexible framework to support further advances in mediation analysis methods.

## Methods

### The standard mediation model, the Sobel test, and LOD drop

The standard mediation model can be described using two linked linear models. For individual *i* = 1, …, *N*, let *y*_*i*_ be the value of the primary outcome or dependent variable, let *m*_*i*_ be the value of the mediator variable, and let *x*_*i*_ be a (scalar) independent variable. Mediation analysis frames the relationships between these variables in terms of the following regressions:

$$\begin{array}{cc} m_{i} & =\mu_{m}+x_{i}\beta_{a}+\epsilon_{i}\;,\\ y_{i} & =\mu_{y}+x_{i}\beta_{c}+m_{i}\beta_{b}+\varepsilon_{i}\;, \end{array}$$

where *μ*_*m*_ and *μ*_*y*_ are intercepts, *ϵ*_*i*_ and *ε*_*i*_ are normally distributed, independent random noise variables, *β*_*a*_ is the effect of *x*_*i*_ on *m*_*i*_, *β*_*b*_ is the effect of *m*_*i*_ on *y*_*i*_, and *β*_*c*_ is the effect of *x*_*i*_ on *y*_*i*_. The effects {*β*_*a*_, *β*_*b*_, *β*_*c*_} correspond to edges {*a*, *b*, *c*} in the DAG in Fig 1. In the language of mediation, *β*_*c*_ describes the direct effect of *x*_*i*_ on *y*_*i*_, whereas the combination of *β*_*a*_ and *β*_*b*_ describe the indirect effect of *x*_*i*_ on *y*_*i*_ via *m*_*i*_.

The Sobel test for mediation focuses on the estimation of the product *β*_*a*_ *β*_*b*_. This product, which provides a single number description of the indirect effect, can take any value from −∞ to ∞ but is zero only when either *β*_*a*_ = 0 or *β*_*b*_ = 0 or both. The traditional implementation of the Sobel test uses classical estimates of *β*_*a*_ *β*_*b*_, and bootstrapping or other approximations [68], to estimate confidence interval for *β*_*a*_ *β*_*b*_ and thereby a *p*-value for *β*_*a*_ *β*_*b*_ ≠ 0. Bayesian implementations of the Sobel test analogously determine a posterior for *β*_*a*_ *β*_*b*_ and thereby a suitable tail probability [27] or Bayes factor [30]. In either implementation, the Sobel test provides a succinct way to quantify the evidence for mediation and uncertainty about the strength and direction of the indirect effect. By contrast, the CS approach [9], and thereby its approximation, the LOD drop method (both defined in the Introduction), is awkwardly constructed [69], lacks power [19, 70], and does not quantify uncertainty; it does, however, take into account the X → Y relationship, potentially enabling discrimination between partial and complete mediation. Note that distinguishing partial and complete mediation is sensitive to misspecification [67] and its value is subject to debate [71].

### Mediation with multiple independent variables

The standard mediation model is trivially adapted to cases where the independent variable is multivariable, that is, of dimension *D* > 1: the scalar predictor *x*_*i*_ and scalar effects *β*_*a*_ and *β*_*c*_ are simply replaced by their *D*-vector counterparts, **x**_*i*_, ***β***_*a*_, ***β***_*c*_. In this case, the CS (or LOD drop) approach to testing mediation may still be applied but, because the product ***β***_*a*_ *β*_*b*_ is a vector, the Sobel test cannot. Sobel test-like procedures have been developed for specific cases, including a bootstrap-based test for when X is multicategorical [63] and a bespoke Bayesian model for when X is a scalar-by-multi-category interaction [28], but these are computationally intensive and do not generalize easily to multivariable X.

### A Bayesian model selection approach to mediation analysis

We propose a Bayesian model selection approach that combines the generality of the CS method with the inferential coherence of the Sobel test. Our approach is most similar to the one in Nuijten et al. 2015 [30], which is also a Bayesian formulation of mediation, but only considers a single independent variable in X. To describe our approach, we first elaborate the standard mediation model to

$$\begin{array}{cc} m_{i} & =\mu_{m}+z_{m,i}^{⊤}\alpha_{m}+\theta_{a}x_{i}^{⊤}\beta_{a}+\epsilon_{i}\;,\\ y_{i} & =\mu_{y}+z_{y,i}^{⊤}\alpha_{y}+\theta_{c}x_{i}^{⊤}\beta_{c}+\theta_{b}m_{i}\beta_{b}+\varepsilon_{i}\;, \end{array}$$

where **x**_*i*_ is a *D*-length column vector, ***β***_*a*_ and ***β***_*c*_ are *D*-length effects vectors, **z**_*m*,*i*_ and **z**_*y*,*i*_ are column vectors encoding any covariates for *m*_*i*_ and *y*_*i*_, ***α***_*m*_ and ***α***_**y**_ are the corresponding covariate effects, $\epsilon_{i}∼N\left(0,\sigma_{m}^{2}/w_{m,i}\right)$ and $\varepsilon_{i}∼N\left(0,\sigma_{y}^{2}/w_{y,i}\right)$ are noise variables with individual-specific weights *w*_**m**, *i*_ and *w*_**y**, *i*_, and ***θ*** = {*θ*_*a*_, *θ*_*b*_, *θ*_*c*_} ∈ {0, 1}^3^ are indicator variables denoting the presence or absence of edges {*a*, *b*, *c*} in the DAG in Fig 1, such that, for example, ***θ*** = {1, 1, 0} denotes complete mediation (*a* and *b* active) with no effect of edge *c*. The parameter space of ***θ*** contains 2^3^ = 8 possible combinations of edges, each of which corresponds to a particular causal model. From a Bayesian perspective, making inferences about the identity of the true causal model is equivalent to calulating the posterior distribution of ***θ***, that is,

$$\begin{array}{c} p(\theta |y,m)\propto p(y,m|\theta )p(\theta )\;, \end{array}$$

where $y={\left\{y_{i}\right\}}_{i=1}^{N}$ and $m={\left\{m_{i}\right\}}_{i=1}^{N}$. This involves calculating a joint likelihood for Y and M given a causal model, p(**y**, **m**|***θ***), and a prior distribution over causal models, p(***θ***). These are described separately below.

### Likelihood and priors conditional on a causal model

The conditional joint likelihood function is given by

$$\begin{array}{cc} m|\theta_{a},\mu_{m},\alpha_{m},\beta_{a},\sigma_{m} & ∼N(\mu_{m}1+Z_{m}\alpha_{m}+\theta_{a}X\beta_{a},\sigma_{m}^{2}W_{m}^{-1})\;,\\ y|m,\theta_{b},\theta_{c},\mu_{y},\alpha_{y},\beta_{b},\beta_{c},\sigma_{y} & ∼N(\mu_{y}1+Z_{y}\alpha_{y}+\theta_{c}X\beta_{c}+\theta_{b}m\beta_{b},\sigma_{y}^{2}W_{y}^{-1})\;, \end{array}$$

where **Z**_**m**_ and **Z**_**y**_ are matrices for the covariates, $W_{m}=\text{diag}\left({\left\{w_{m,i}\right\}}_{i=1}^{N}\right)$ and $W_{y}=\text{diag}\left({\left\{w_{y,i}\right\}}_{i=1}^{N}\right)$ are diagonal matrices of observation weights. By default, **Z**_**m**_ = **Z**_**y**_ = **0**, and **W**_**m**_ = **W**_**y**_ = **I**. We assume that *y*_*i*_ and *m*_*i*_ have each been standardized to have zero mean and unit variance.

Conjugate priors are specified for the following variables:

$$\begin{array}{cccc} \mu_{m}|\sigma_{m} & ∼N(0,\sigma_{m}^{2}\tau_{\mu_{m}}^{2})\;, & \mu_{y}|\sigma_{y} & ∼N(0,\sigma_{y}^{2}\tau_{\mu_{y}}^{2})\;,\\ \alpha_{m}|\sigma_{m} & ∼N(0,\sigma_{m}^{2}\tau_{Z_{m}}^{2}I)\;, & \alpha_{y}|\sigma_{y} & ∼N(0,\sigma_{y}^{2}\tau_{Z_{y}}^{2}I)\;,\\ \beta_{a}|\sigma_{m} & ∼N(0,\sigma_{m}^{2}\phi_{a}^{2}I)\;, & \beta_{c}|\sigma_{y} & ∼N(0,\sigma_{y}^{2}\phi_{c}^{2}I)\;,\\ & & \beta_{b}|\sigma_{y} & ∼N(0,\sigma_{y}^{2}\phi_{b}^{2})\;,\\ \sigma_{m}^{-2} & ∼\text{Ga}(0.5\kappa_{m},0.5\lambda_{m})\;, & \sigma_{y}^{-2} & ∼\text{Ga}(0.5\kappa_{y},0.5\lambda_{y})\;. \end{array}$$

These variables can be integrated from the likelihood due to conjugacy, giving a closed form expression for the marginal joint likelihood function:

$$\begin{array}{cc} m|\theta_{a} & ∼t_{\kappa_{m}}\left(0,\lambda_{m}\left[W^{-1}+X_{m}V_{m}X_{m}^{T}\right]\right)\;,\\ y|m,\theta_{b},\theta_{c} & ∼t_{\kappa_{y}}\left(0,\lambda_{y}\left[W^{-1}+X_{y}V_{y}X_{y}^{T}\right]\right)\;, \end{array}$$

where $X_{m}$ and $X_{y}$ are concatenated design matrices and **V**_**m**_ and **V**_**y**_ are prior covariance matrices for the effect variables, specifically,

$$\begin{array}{cccc} X_{m} & =[\begin{array}{ccc} 1 & Z_{m} & \theta_{a}X \end{array}]\;, & V_{m} & =[\begin{array}{ccc} \tau_{\mu }^{2} & 0 & 0\\ 0 & \tau_{Z}^{2}I & 0\\ 0 & 0 & \phi_{a}^{2}I \end{array}]\;,\\ X_{y} & =[\begin{array}{cccc} 1 & Z_{y} & \theta_{c}X & \theta_{b}m \end{array}]\;, & V_{y} & =[\begin{array}{cccc} \tau_{\mu }^{2} & 0 & 0 & 0\\ 0 & \tau_{Z}^{2}I & 0 & 0\\ 0 & 0 & \phi_{c}^{2}I & 0\\ 0 & 0 & 0 & \phi_{b}^{2} \end{array}]\;. \end{array}$$

This marginal joint likelihood is evaluated for all causal models ***θ*** = (*θ*_*a*_, *θ*_*b*_, *θ*_*c*_), given prior hyperparameters ***κ*** = (*κ*_**m**_, *κ*_**y**_), **λ** = (λ_**m**_, λ_**y**_), $\tau_{\mu }^{2}=\left(\tau_{\mu_{m}}^{2},\tau_{\mu_{y}}^{2}\right)$, $\tau_{Z}^{2}=\left(\tau_{Z_{m}}^{2},\tau_{Z_{y}}^{2}\right)$, and $\phi^{2}=\left(\phi_{a}^{2},\phi_{b}^{2},\phi_{c}^{2}\right)$. Non-informative priors are used for the scale of the data [***κ*** = **λ** = (0.001, 0.001)], the location of the intercept [***τ***_*μ*_ = (1000, 1000)], and covariate effects [***τ***_**Z**_ = (1000, 1000)]. The hyperparameter ***ϕ***^2^ controls the prior effect size of each edge, relative to error, on **m** or **y**. We set ***ϕ***^2^ = (1, 1, 1) by default, such that effect sizes for all edges are equal and relatively large *a priori*.

### Priors for the causal models

In our framework, a prior probability is assigned to each possible causal model. The default model prior places uniform prior probability across the eight models for which edge *b* is either absent or present as M→Y (no reactive models). That is,

$$\begin{array}{c} \text{Default:}\;\text{p}\left(\theta \right)\propto 1\;\text{for}\;\theta \in {\left\{0,1\right\}}^{3}\;, \end{array}$$

such that the prior probability for each of ML1–8 in Fig 1 is $\frac{1}{8}$. We also consider two other prior specifications: the reduced model prior, which additionally assumes that edge *c* is present, that is,

$$\begin{array}{c} \text{Reduced:}\;\text{p}\left(\theta \right)\propto 1\;\text{for}\;\left(\theta_{a},\theta_{b}\right)\in {\left\{0,1\right\}}^{2}\;\text{and}\;\theta_{c}=1\;, \end{array}$$

such that the prior probability for each of ML4–8 is $\frac{1}{4}$; and the expanded model prior, which allows models with a reversed edge *b* (*i.e.*, Y→M) and is described in more detail below. In our framework and using our bmediatR software, it is possible to specify priors as any distinct set of allowable causal models, or, with greater granularity, as relative weights across the set of models. This feature provides a foundation that could accommodate empirically-determined prior weights out-of-the-box.

### Empirical effect size priors

As an alternative to the default pure prior specification of the effect size hyperparameter ***ϕ***^2^, we consider the following empirical approach. Define the proportion of variation explained by the explanatory variable on each outcome as $\text{PVE}=1-\frac{\sum_{i}^{n}e_{i}^{2}|M_{A}}{\sum_{i}^{n}e_{i}^{2}|M_{0}}$, where $\sum_{i}^{n}e_{i}^{2}|M_{A}$ is the sum of squared residuals when an edge is present and $\sum_{i}^{n}e_{i}^{2}|M_{0}$ is the null sum of squared residuals when there is no relationship. For *a*, M is regressed onto X for *M*_*A*_ and an intercept-only model for *M*_0_. For *b*, Y is regressed onto M for *M*_*A*_ and an intercept-only model for *M*_0_. For *c*, Y is regressed onto X for *M*_*A*_ and an intercept-only model for *M*_0_. The hyperparameters in ***ϕ***^2^ are then input as their ratio to noise variation as $\hat{\phi^{2}}=\frac{\hat{\text{PVE}}}{1-\hat{\text{PVE}}}$.

### Allowing reverse causality between M and Y

The bmediatR software can also model reverse causality between the candidate mediator and trait (*i.e.*, Y→M). This can be described using a third state for *θ*_*b*_, denoted by *θ*_*b*_ = *, giving a total of twelve possible causal models encoded by ***θ***. These include the eight causal models previously described, as well as four additional reverse causality (or “reactive”) cases, given by (*θ*_*a*_, *θ*_*b*_ = *, *θ*_*c*_), where models with *θ*_*b*_ = * are specified with the roles of all Y- and M-related variable above interchanged (explicit formulae given in S1 Text). Although any set of relative prior probabilities could be used, we here consider a simple prior (the expanded prior) that is uniform over the 12 causal models, that is,

$$\begin{array}{c} \text{Expanded:}\;\text{p}\left(\theta \right)\propto 1\;\text{for}\;\left(\theta_{a},\theta_{c}\right)\in {\left\{0,1\right\}}^{2}\;\text{and}\;\theta_{b}\in \left\{0,1,*\right\}\;, \end{array}$$

such that the prior probability for each of ML1–12 in Fig 1 is $\frac{1}{12}$.

Note that two pairs of these twelve causal models are not identifiable from one another: ***θ*** = (0, 1, 0) is indistinguishable from ***θ*** = (0, *, 0); and ***θ*** = (1, 1, 1) (partial mediation) is indistinguishable from ***θ*** = (1, *, 1) (reactive partial mediation). The marginal joint likelihood functions for these pairs of models are identical, and these causal relationships cannot be distinguished without additional information. Despite this limitation, we include all twelve causal relationships as possibilities in our expanded model prior, and emphasize that inference about the direction of causality in these cases depends entirely on prior information and assumptions.

### Bayesian network analysis

We compared our approach with a frequentist method that can also explore the same DAGs, namely Bayesian network analysis, as implemented in the R package bnlearn [38]. The pre-set model priors of bmediatR were replicated in bnlearn by forcibly excluding and including edges through “blacklisting” and “whitelisting”, respectively. To match bmediatR’s default model priors, Y→X, M→X, and Y→M are all blacklisted. For bmediatR’s reduced model priors, Y→X, M→X, and Y→M are all again blacklisted, and X→Y is whitelisted. Lastly, for bmediatR’s expanded model priors, only Y→X and M→X are blacklisted. Although this implementation of model priors in bnlearn replicates bmediatR’s ability to exclude likelihoods using 0 weights, it does not permit granular weights between 0 and 1. The best model was learned from the data using the Tabu search greedy algorithm [72], and this was reported as a point estimate for the causal model.

### Simulation of QTL data

To simulate data with an exogenous variable representing genetic variation (*e.g.*, SNPs, founder haplotypes), we expanded our previous approach for simulating QTL in the Collaborative Cross mouse population [73]. The model describes relationships between two traits with shared genetic drivers, representing either co-local or mediation.

We simulate a single trait with a single QTL based on a simple linear model:

$$\begin{array}{c} y_{\text{sim}}=X\beta +\varepsilon \;, \end{array}$$ (1)

where **y** is the trait vector, **X** is the design matrix of the genetic information (*e.g.*, SNP allele count, founder haplotype count), ***β*** is the genetic effects vector (*e.g.*, SNP allele effect, haplotype effects), and ***ε*** is a random noise vector. We scale ***β*** and ***ε*** such that their relative contributions to **y**_sim_ match a specified proportion of variation of **y**_sim_ explained by **X*β***, *i.e.*, the effect size of X on Y. If an initial genetic effect vector (***β***_raw_) is not specified, we sample one according to ***β***_raw_ ∼ N(**0**, **I**), where **I** is the identity matrix of rank equal to length of ***β***. An initial noise vector is also sampled as ***ε***_raw_ ∼ N(**0**, **I**_*n*_) where **I**_*n*_ is the identity matrix of rank *n*, the number of rows of **X**. For a specified effect size *ϕ*^2^ (ranging from 0 to 1), we define scaling factors for ***β***_raw_ and ***ε***_raw_ to approximate the desired effect size of X on Y:

$$\begin{array}{c} s_{\beta }={\left(\frac{n\phi^{2}}{\left(n-1\right)V\left(X\beta_{\text{raw}}\right)}\right)}^{\frac{1}{2}}\;,\;\text{and}\;s_{\varepsilon }={\left(\frac{n(1-\phi^{2})}{\left(n-1)V(\varepsilon_{\text{raw}}\right)}\right)}^{\frac{1}{2}}\;, \end{array}$$

where *V*() returns the population variance of its argument. The effects and noise vectors are then scaled: ***β*** = *s_β_ **β***_raw_ and ***ε*** = *s_ε_ **ε***_raw_.

For the co-local simulations, both the dependent variable **y**_sim_ and the mediator variable **m**_sim_ are simulated as in Eq 1 with the same ***β***_raw_ but independent draws of ***ε***_raw_. For the complete mediation simulations, **m**_sim_ is simulated according to Eq 1, and the dependent variable **y**_sim_ is simulated based on a linear model with the mediator as a predictor:

$$\begin{array}{c} y_{\text{sim}}=m_{\text{sim}}\beta_{m}+\epsilon \;, \end{array}$$

where ***β***_**m**_ and ***ϵ*** are derived from scaled raw variables just as ***β*** and ***ε*** were in Eq 1 in order to strictly control how M contributes to variation in Y (*i.e.*, effect size of M on Y).

The partial mediation simulations are necessarily more complicated. The mediator **m**_sim_ is again simulated according to Eq 1. The linear model for **y**_sim_ includes effects from both X and M:

$$\begin{array}{c} y_{\text{sim}}=X\beta +m_{\text{sim}}\beta_{m}+\epsilon \;, \end{array}$$

where ***β*** is the direct effect of X on Y and ***β***_**m**_ is the indirect effect. ***β***, ***β***_**m**_, and ***ϵ*** were derived from scaled raw variables as before in order to strictly control how X and M together contribute to variation in Y (*i.e.*, combined effect size on Y). Reactive partial and reactive complete mediation were simulated exactly as partial and complete mediation, but with **y**_sim_ and **m**_sim_ swapped in the mediation procedures.

For the large-scale simulations of a single locus (Figs 2, 4 and S3 Fig, S1, S2, S4 and S5 Figs), **X** represented balanced functional allele counts (SNP or founder haplotypes) for 200 individuals. One hundred simulations were performed for each combination of effect size on M and Y (ranging from 0.05 to 0.95 at regular intervals of 0.05) for each data-generating model (co-local, partial mediation, complete mediation, reactive complete mediation, and reactive partial mediation). For the partial mediation simulations, the ratio of direct and indirect effect was fixed at 1 (b:c = 1). For each data-generating model, Bayesian model selection, Bayesian network analysis, Sobel test (if **X** represented a SNP), LOD drop, and IV analysis were summarized.

For the simulations comparing the methods using ROC curves (Fig 3 and S7 Fig), we used a balanced bi-allelic **X**, but for only 24 individuals. For two effect size settings for each data-generating model, we peformed 5,000 simulations. For bmediatR and bnlearn, true positive and false positive rates were based on the sum of complete and partial mediation probabilities.

For the simulations demonstrating multi-allelic QTL analysis with mediation in Fig 5, the design matrix **X** represents additive effects of founder haplotypes at a randomly sampled genetic locus from a population of 192 DO mice [5]. The underlying simulated genetic effect was from a bi-allelic SNP, with each allele present in four of the founder strains.

### Diversity outbred mouse data

The DO mouse data represent 192 animals [5] with both gene expression and protein abundance from bulk liver tissue, representing a subset of a larger cohort of 850 animals [74]. Approximately equal numbers of males and females are present as well as animals on standard and high fat diets. The data are publicly available for interactive analysis as a QTL Viewer (https://github.com/churchill-lab/qtlapi) which also allows a bulk download of the underlying R data files (https://qtlviewer.jax.org/viewer/SvensonHFD).

#### QTL analysis

Samples of liver tissue were collected and processed for quantitative mass-spectrometry as previously described [5]. Estimation and normalization of the protein abundance data from component quantitative peptide data and subsequent QTL analysis have been previously described [13]. Genetic mapping was based on final quantities output from the rank-based inverse normal transformation (RINT) [75].

The examples related to the distal pQTL of *Snx4* and *Tubg1* were previously identified [13]. QTL were mapped using the following linear mixed effect model:

$$\begin{array}{c} {\text{trait}}_{i}=\text{intercept}+{\text{covariates}}_{i}+{\text{QTL}}_{i,m}+u_{i}+\varepsilon_{i}\;, \end{array}$$ (2)

where trait_*i*_ is the phenotype of interest (abuandance of a protein) for individual *i*, intercept is a shared intercept that represents the mean trait value, covariates_*i*_ is the effect of known covariates on individual *i*, QTL_*i*,*m*_ is the effect of genetic variation at genomic interval *m* on individual *i*, *u*_*i*_ is a random error term that accounts for the similarity of individual *i* to other samples proportional to overall genetic relatedness (kinship effect), and *ε*_*i*_ is the unstructured error on individual *i*. For the QTL term: ${\text{QTL}}_{i,m}=d_{i}^{T}\beta_{\text{QTL}}$, where **d**_*i*_ is the vector of additive dosages of founder haplotypes for the genomic interval *m* and ***β***_QTL_ are the haplotype effects at the putative QTL, estimated as fixed effects. The random error terms are modeled as **u** ∼ N(**0**, **K***τ*^2^) and *ε*_*i*_ ∼ N(0, *σ*^2^), where **K** is the realized genetic relationship matrix excluding information from the chromosome of the current genomic interval *m* (“loco” method), and *τ*^2^ and *σ*^2^ are variance components for *u*_*i*_ and *ε*_*i*_, respectively. Covariates adjusted for include sex, diet, and DO litter (2 levels). Eq 2 is fit at genomic intervals spanning the entire chromosome, comprising a QTL genome scan. Models were fit using the qtl2 R package [76]. For the variant association performed for the *Snx4* and *Snx7* pQTL, Eq 2 was used, but with the QTL_*i*,*m*_ adjusted. Instead of representing the effects of doses of founder haplotypes, variant allele dosages were imputed based on the founder haplotype dosages and the variant genotype-to-founder strains distribution (SQLite variant database: https://doi.org/10.6084/m9.figshare.5280229.v3).

#### Haplotype effect estimation

To compare the similarity of the genetic effects on M and Y, the correlation coefficients between haplotype effects of QTL were calculated. To stabilize haplotype effect estimates, instead of the fixed effect estimate from Eq 2, they were estimated as best linear unbiased predictions (BLUPs) in the qtl2 R package.

#### Modeling the allelic series of QTL with TIMBR

We modeled the allelic series at the pQTL for SNX4 and TUBG1 using a Bayesian hierarchical model as implemented in TIMBR [40]. The model is roughly equivalent to Eq 2, with the kinship effect excluded in order to make the computation feasible. A Chinese restaurant process prior was used for the allelic series, as well as the prescribed shape and rate parameters of 1 and ≈2.33, respectively, for the concentration parameter, which favors smaller numbers of functional alleles with low variance.

TIMBR models founder haplotype uncertainty at the QTL based on the 36 genotype states. We reconstructed founder haplotype probabilities from the available genotype array data (https://www.jax.org/research-and-faculty/genetic-diversity-initiative/tools-data/diversity-outbred-database), which represented 187 of the 192 mice.

### Human cell line data

The genotype data from 119 Yoruba LCLs represent variants from the intersection of HapMap2 and HapMap3, coded as the major allele count for each SNP [43]. The RNA-seq data [44] were adjusted with WASP [77] and normalized [45], representing 69 LCLs [43]. Both genotype and RNA-seq data are publicly available for download (http://eqtl.uchicago.edu/jointLCL/). The DNase-seq data for the 69 cell lines with RNA-seq data were used as previously processed [46]. The overlap of samples across genotypes, RNA-seq, and DNase-seq was 63 LCLs.

### Chromatin accessibility and expression QTL analysis

To identify candidates for co-mapping eQTL and cQTL, we correlated the expression of gene with chromatin peaks within 80 Kbp of the gene start. QTL mapping was then performed for genes and chromatin peaks that had correlations > 0.5. Mapping was performed by regressing a trait (gene expression or chromatin accessibility) onto the genotypes of individual SNPs located on the gene’s chromosome, and compared with a null model with no genotype term. QTL were called for SNPs that produced a -log10 *p*-value > 8, representing a stringent threshold for 63 individuals. Gene-chromatin peak pairs were then filtered to those with an eQTL and cQTL co-mapping to the same SNP. For each passing gene, all chromatin peaks on the gene’s chromosome were tested as candidate mediators using both Bayesian model selection, with default prior settings, and the Sobel test.

## Software

All statistical analyses were conducted with the R statistical programming language [78]. The associated R package, bmediatR, was used for all analyses, and is available on GitHub at https://github.com/wesleycrouse/bmediatR and a frozen version as S1 File.

## Supporting information

S1 Fig Performance of Bayesian model selection in simulated QTL data from non-reactive models across varying priors for effect size and allowable models. Data for 200 individuals were simulated according to (a) co-local, (b) partial mediation, and (c) complete mediation models from a balanced bi-allelic SNP. DAGs indicate the model used to simulate the data. Heat maps represent the mean posterior probability associated with each inferred model for a range of fixed settings of the model parameters as indicated on x- and y-axes, each simulated 100 times. Bayesian model selection was performed using the (left) default effect size priors (50% for a, b, and c) and (right) empirical effect size priors. Model priors were varied, represented as the rows within an individual panel. Empty squares represent posterior model categories not evaluated based on the set of allowable models encoded in the model priors. See S2 Fig for results from reactive model simulations.
(PDF)

S2 Fig Performance of Bayesian model selection in simulated QTL data from reactive models across varying priors for effect size and allowable models. Data for 200 individuals were simulated according to (a) reactive partial mediation and (b) reactive complete mediation models from a balanced bi-allelic SNP. DAGs indicate the model used to simulate the data. Heat maps represent the mean posterior probability associated with each inferred model for a range of fixed settings of the model parameters as indicated on x- and y-axes, each simulated 100 times. Bayesian model selection was performed using the (left) default effect size priors (50% for a, b, and c) and (right) empirical effect size priors. Model priors were varied, represented as the rows within an individual panel. Empty squares represent posterior model categories not evaluated based on the set of allowable models encoded in the model priors. See S1 Fig for results from non-reactive model simulations.
(PDF)

S3 Fig Performance of Bayesian model selection (bmediatR) compared with a Bayesian network analysis (bnlearn) in simulated data with a binary exogenous variable. Data for 200 individuals were simulated according to (a) co-local, (b) partial mediation, and (c) complete mediation. DAGs indicate the model used to simulate the data. Heat maps for Bayesian model selection represent the mean posterior probability associated with each inferred model for a range of fixed settings of the model parameters as indicated on x- and y-axes, each simulated 100 times. Default prior settings were used. Heat maps for Bayesian network analysis represent the best bnlearn model probability across 100 simulations. See S1 and S2 Figs for Bayesian model selection results using empirical effect size priors and non-default model priors, including reactive. See S4 and S5 Figs for similar results from Bayesian network analysis.
(PDF)

S4 Fig Performance of Bayesian network analysis, Sobel test, LOD drop, and IV regression in simulated QTL data from non-reactive models. Data for 200 individuals were simulated according to (a) co-local, (b) partial mediation, and (c) complete mediation models from a balanced bi-allelic SNP. DAGs indicate the model used to simulate the data. Heat maps for Bayesian network analysis represent the best bnlearn model probability across 100 simulations for a range of fixed settings of the model parameters as indicated on x- and y-axes. Heat maps for the Sobel test and IV regression represent false positive probability for co-local simulations and power for mediation simulations. Heat maps for LOD drop represent mean LOD drop, scaled to the proportion of the simulated QTL’s LOD score. Model priors were varied, represented as the rows within an individual panel. Empty squares represent posterior model categories not evaluated based on the set of allowable models encoded in the model priors. See S5 Fig for results from reactive model simulations.
(PDF)

S5 Fig Performance of Bayesian network analysis, Sobel test, LOD drop, and IV regression in simulated QTL data from reactive models. Data for 200 individuals were simulated according to (a) reactive partial mediation and (b) reactive complete mediation models from a balanced bi-allelic SNP. DAGs indicate the model used to simulate the data. Heat maps for Bayesian network analysis represent the best bnlearn model probability across 100 simulations for a range of fixed settings of the model parameters as indicated on x- and y-axes. Heat maps for the Sobel test and IV regression represent false positive probability for co-local simulations and power for mediation simulations. Heat maps for LOD drop represent mean LOD drop, scaled to the proportion of the simulated QTL’s LOD score. Model priors were varied, represented as the rows within an individual panel. Empty squares represent posterior model categories not evaluated based on the set of allowable models encoded in the model priors. See S4 Fig for results from non-reactive model simulations.
(PDF)

S6 Fig Example scenarios comparing inference from Bayesian model selection (bmediatR) to a Bayesian network analysis (bnlearn) in simulated data with a binary exogenous variable. (a) Data for 200 individuals were simulated according to partial mediation. The DAG represents the partial mediation model used to simulate the data. Heat maps for Bayesian model selection represent the mean posterior probability associated with each inferred model for a range of fixed settings of the model parameters as indicated on x- and y-axes, each simulated 100 times. Default prior settings were used. Heat maps for Bayesian network analysis represent the best bnlearn model probability, across 100 simulations. Two effect size settings are marked and explored further: (b) + and (c) ×. For each scenario (b-c), bmediatR returned posterior model probabilities (left) and bnlearn returned best models (right) for 10,000 simulations. Best models from bnlearn are represented as stacked bar plots for each model, with solid bars indicating the number of simulations for which the model was selected as best model and transparent bars indicate the number of simulations for which it was not selected. (d) Posterior model probabilities for a simulated mediator that bnlearn classified correctly as partial (left) and incorrectly as co-local (right).
(PDF)

S7 Fig Performance of Bayesian model selection compared with other methods in distinguishing partial mediation from (a-b) co-local and (c-d) non-mediation. True positive rates (power) and false positive rates were estimated from 5,000 simulations of 24 individuals according to a balanced bi-allelic variant X. Results are shown for data simulated with both (a-c) small genetic effects (X → M: 10%, M → Y: 5%, X → Y: 5%) and (b-d) large genetic effects (X → M: 50%, M → Y: 25%, X → Y: 25%). Diagonal dashed line is included for reference, representing a classifier with no ability to distinguish complete mediation from co-local or non-mediation. See Fig 3 for methods’ performance in distinguishing complete mediation from co-local and non-mediation.
(PDF)

S8 Fig Modeling the allelic series of pQTL for *Snx4*, *Tubg1*, and related genes identified through mediation. The allelic series were modeled with TIMBR. (a) Posterior haplotype effects and allelic series for the *Snx4* distal pQTL and the local pQTL for its mediator, *Snx7*. Haploytpe effects are represented as histograms of the posterior samples. Allelic series are represented as circos plots where the opacity of the links represent how often TIMBR assigned founder haplotypes to the same functional allele. (b) Posterior haplotype effects and allelic series for the *Tubg1* and *Tubgcp2* distal pQTL and the local pQTL for their candidate mediators, *Tubgcp3* and *Naxd*. The posterior expected number of functional alleles, *k*, is included for each pQTL.
(PDF)

S1 Table Features of the evaluated causal inference methods. (PDF)

S1 Text Joint likelihood for causal models in the reactive mode. (PDF)

S1 File A frozen version (0.1.2) of bmediatR, the R package implementation of Bayesian model selection. (GZ)

## References

[1] Judd CM, Kenny DA. Data Analysis in Social Psychology: Recent and Recurring Issues. In: Fiske ST, Gilbert DT, Lindzey G, editors. Handbook of Social Psychology. Hoboken, N.J.: American Cancer Society; 2010. Available from: https://onlinelibrary.wiley.com/doi/abs/10.1002/9780470561119.socpsy001004.

[2] MacKinnon DP, Fairchild AJ, Fritz MS. Mediation Analysis. Annual Review of Psychology. 2007;58(1):593–614. doi: 10.1146/annurev.psych.58.110405.085542 16968208 PMC2819368

[3] Raulerson CK, Ko A, Kidd JC, Currin KW, Brotman SM, Cannon ME, et al. Adipose Tissue Gene Expression Associations Reveal Hundreds of Candidate Genes for Cardiometabolic Traits. The American Journal of Human Genetics. 2019;105(4):773–787. doi: 10.1016/j.ajhg.2019.09.001 31564431 PMC6817527

[4] Yao DW, O’Connor LJ, Price AL, Gusev A. Quantifying genetic effects on disease mediated by assayed gene expression levels. Nature Genetics. 2020;52(6):626–633. doi: 10.1038/s41588-020-0625-2 32424349 PMC7276299

[5] Chick JM, Munger SC, Simecek P, Huttlin EL, Choi K, Gatti DM, et al. Defining the consequences of genetic variation on a proteome-wide scale. Nature. 2016;534(7608):500–5. doi: 10.1038/nature18270 27309819 PMC5292866

[6] Keele GR, Quach BC, Israel JW, Chappell GA, Lewis L, Safi A, et al. Integrative QTL analysis of gene expression and chromatin accessibility identifies multi-tissue patterns of genetic regulation. PLOS Genetics. 2020;16(1):e1008537. doi: 10.1371/journal.pgen.1008537 31961859 PMC7010298

[7] Judd CM, Kenny DA. Process Analysis: Estimating Mediation in Treatment Evaluations. Evaluation Review. 1981;5(5):602–619. doi: 10.1177/0193841X8100500502

[8] Pearl J. Interpretation and identification of causal mediation. Psychological Methods. 2014;19(4):459–481. doi: 10.1037/a0036434 24885338

[9] Baron RM, Kenny DA. The moderator–mediator variable distinction in social psychological research: Conceptual, strategic, and statistical considerations. Journal of Personality and Social Psychology. 1986;51(6):1173–1182. doi: 10.1037/0022-3514.51.6.1173 3806354

[10] Sobel ME. Asymptotic Confidence Intervals for Indirect Effects in Structural Equation Models. Sociological Methodology. 1982;13(1982):290. doi: 10.2307/270723

[11] Preacher KJ, Hayes AF. Asymptotic and resampling strategies for assessing and comparing indirect effects in multiple mediator models. Behavior Research Methods. 2008;40(3):879–891. doi: 10.3758/BRM.40.3.879 18697684

[12] Collaborative Cross Consortium. The genome architecture of the Collaborative Cross mouse genetic reference population. Genetics. 2012;190(2):389–401. doi: 10.1534/genetics.111.132639 22345608 PMC3276630

[13] Keele GR, Zhang T, Pham DT, Vincent M, Bell TA, Hock P, et al. Regulation of protein abundance in genetically diverse mouse populations. Cell Genomics. 2021;1(1):100003. doi: 10.1016/j.xgen.2021.100003 PMC9536773 36212994

[14] Churchill G, Gatti D, Munger S, Svenson K. The Diversity outbred mouse population. Mammalian Genome. 2012;23:713–8. doi: 10.1007/s00335-012-9414-2 22892839 PMC3524832

[15] Keller MP, Gatti DM, Schueler KL, Rabaglia ME, Stapleton DS, Simecek P, et al. Genetic Drivers of Pancreatic Islet Function. Genetics. 2018;209(1):335–356. doi: 10.1534/genetics.118.300864 29567659 PMC5937189

[16] Skelly DA, Czechanski A, Byers C, Aydin S, Spruce C, Olivier C, et al. Mapping the Effects of Genetic Variation on Chromatin State and Gene Expression Reveals Loci That Control Ground State Pluripotency. Cell Stem Cell. 2020; p. 1–11. doi: 10.1016/j.stem.2020.07.005 32795400 PMC7484384

[17] Zhong W, Spracklen CN, Mohlke KL, Zheng X, Fine J, Li Y. Multi-SNP mediation intersection-union test. Bioinformatics. 2019;35(22):4724–4729. doi: 10.1093/bioinformatics/btz285 31099385 PMC6853702

[18] Liu Z, Shen J, Barfield R, Schwartz J, Baccarelli AA, Lin X. Large-Scale Hypothesis Testing for Causal Mediation Effects with Applications in Genome-wide Epigenetic Studies. Journal of the American Statistical Association. 2021; p. 1–39. 10.1080/01621459.2021.1914634 PMC9385159 35989709

[19] MacKinnon DP, Lockwood CM, Hoffman JM, West SG, Sheets V. A comparison of methods to test mediation and other intervening variable effects. Psychological Methods. 2002;7(1):83–104. doi: 10.1037/1082-989x.7.1.83 11928892 PMC2819363

[20] Katan M. Apolipoprotein E isoforms, serum cholesterol, and cancer. The Lancet. 1986;327(8479):507–508. doi: 10.1016/S0140-6736(86)92972-7 2869248

[21] Didelez V, Sheehan N. Mendelian randomization as an instrumental variable approach to causal inference. Statistical methods in medical research. 2007;16(4):309–30. doi: 10.1177/0962280206077743 17715159

[22] Voight BF, Peloso GM, Orho-Melander M, Frikke-Schmidt R, Barbalic M, Jensen MK, et al. Plasma HDL cholesterol and risk of myocardial infarction: a mendelian randomisation study. The Lancet. 2012;380(9841):572–580. doi: 10.1016/S0140-6736(12)60312-2 22607825 PMC3419820

[23] Davies NM, Holmes MV, Davey Smith G. Reading Mendelian randomisation studies: a guide, glossary, and checklist for clinicians. BMJ. 2018;362:k601. doi: 10.1136/bmj.k601 30002074 PMC6041728

[24] Carter AR, Sanderson E, Hammerton G, Richmond RC, Davey Smith G, Heron J, et al. Mendelian randomisation for mediation analysis: current methods and challenges for implementation. European Journal of Epidemiology. 2021;36(5):465–478. doi: 10.1007/s10654-021-00757-1 33961203 PMC8159796

[25] Burgess S, Daniel RM, Butterworth AS, Thompson SG, the EPIC-InterAct Consortium. Network Mendelian randomization: using genetic variants as instrumental variables to investigate mediation in causal pathways. International Journal of Epidemiology. 2015;44(2):484–495. doi: 10.1093/ije/dyu176 25150977 PMC4469795

[26] Burgess S, Thompson DJ, Rees JMB, Day FR, Perry JR, Ong KK. Dissecting Causal Pathways Using Mendelian Randomization with Summarized Genetic Data: Application to Age at Menarche and Risk of Breast Cancer. Genetics. 2017;207(2):481–487. doi: 10.1534/genetics.117.300191 28835472 PMC5629317

[27] Yuan Y, MacKinnon DP. Bayesian mediation analysis. Psychological Methods. 2009;14(4):301–322. doi: 10.1037/a0016972 19968395 PMC2885293

[28] Oreper D, Schoenrock SA, McMullan R, Ervin R, Farrington J, Miller DR, et al. Reciprocal F1 Hybrids of Two Inbred Mouse Strains Reveal Parent-of-Origin and Perinatal Diet Effects on Behavior and Expression. G3: Genes, Genomes, Genetics. 2018;8(11):3447–3468. doi: 10.1534/g3.118.200135 30171036 PMC6222572

[29] Song Y, Zhou X, Zhang M, Zhao W, Liu Y, Kardia SLR, et al. Bayesian shrinkage estimation of high dimensional causal mediation effects in omics studies. Biometrics. 2020;76(3):700–710. doi: 10.1111/biom.13189 31733066 PMC7228845

[30] Nuijten MB, Wetzels R, Matzke D, Dolan CV, Wagenmakers EJ. A default Bayesian hypothesis test for mediation. Behavior Research Methods. 2015;47(1):85–97. doi: 10.3758/s13428-014-0470-2 24903686

[31] Nagarajan R, Scutari M, Lèbre S. Bayesian Networks in R, with Applications in Systems Biology. New York: Springer; 2013.

[32] Howey R, Shin SY, Relton C, Davey Smith G, Cordell HJ. Bayesian network analysis incorporating genetic anchors complements conventional Mendelian randomization approaches for exploratory analysis of causal relationships in complex data. PLOS Genetics. 2020;16(3):e1008198. doi: 10.1371/journal.pgen.1008198 32119656 PMC7067488

[33] Howey R, Clark AD, Naamane N, Reynard LN, Pratt AG, Cordell HJ. A Bayesian network approach incorporating imputation of missing data enables exploratory analysis of complex causal biological relationships. PLOS Genetics. 2021;17(9):e1009811. doi: 10.1371/journal.pgen.1009811 34587167 PMC8504979

[34] Viñuela A, Brown AA, Fernandez J, Hong MG, Brorsson CA, Koivula RW, et al. Genetic analysis of blood molecular phenotypes reveals regulatory networks affecting complex traits: a DIRECT study. medRxiv. 2021. 10.1038/s41467-023-40569-3 PMC10442420 37604891

[35] Neto EC, Keller MP, Attie AD, Yandell BS. Causal graphical models in systems genetics: A unified framework for joint inference of causal network and genetic architecture for correlated phenotypes. The Annals of Applied Statistics. 2010;4(1):320–339. doi: 10.1214/09-aoas288 21218138 PMC3017382

[36] Hageman RS, Leduc MS, Korstanje R, Paigen B, Churchill GA. A Bayesian framework for inference of the genotype–phenotype map for segregating populations. Genetics. 2011;187(4):1163–1170. doi: 10.1534/genetics.110.123273 21242536 PMC3070524

[37] Fox J, Kleiber C, Zeileis A. ivreg: Instrumental-Variables Regression by ‘2SLS’, ‘2SM’, or ‘2SMM’, with Diagnostics; 2021. Available from: https://CRAN.R-project.org/package=ivreg.

[38] Scutari M. Learning Bayesian Networks with the bnlearn R Package. Journal of Statistical Software. 2010;35(3):1–22. doi: 10.18637/jss.v035.i0321603108

[39] Worby CA, Dixon JE. Sorting out the cellular functions of sorting nexins. Nature Reviews Molecular Cell Biology. 2002;3(12):919–931. doi: 10.1038/nrm974 12461558

[40] Crouse WL, Kelada SNP, Valdar W. Inferring the Allelic Series at QTL in Multiparental Populations. Genetics. 2020;216(4):957–983. doi: 10.1534/genetics.120.303393 33082282 PMC7768242

[41] Oakley BR, Paolillo V, Zheng Y. *γ*-Tubulin complexes in microtubule nucleation and beyond. Molecular Biology of the Cell. 2015;26(17):2957–2962. doi: 10.1091/mbc.E14-11-1514 26316498 PMC4551311

[42] Farache D, Emorine L, Haren L, Merdes A. Assembly and regulation of *γ*-tubulin complexes. Open Biology. 2018;8(3):170266. doi: 10.1098/rsob.170266 29514869 PMC5881034

[43] Li YI, van de Geijn B, Raj A, Knowles DA, Petti AA, Golan D, et al. RNA splicing is a primary link between genetic variation and disease. Science. 2016;352(6285):600–604. doi: 10.1126/science.aad9417 27126046 PMC5182069

[44] Pickrell JK, Marioni JC, Pai AA, Degner JF, Engelhardt BE, Nkadori E, et al. Understanding mechanisms underlying human gene expression variation with RNA sequencing. Nature. 2010;464(7289):768–772. doi: 10.1038/nature08872 20220758 PMC3089435

[45] Degner JF, Pai AA, Pique-Regi R, Veyrieras JB, Gaffney DJ, Pickrell JK, et al. DNase I sensitivity QTLs are a major determinant of human expression variation. Nature. 2012;482(7385):390–394. doi: 10.1038/nature10808 22307276 PMC3501342

[46] Grubert F, Zaugg JB, Kasowski M, Ursu O, Spacek DV, Martin AR, et al. Genetic Control of Chromatin States in Humans Involves Local and Distal Chromosomal Interactions. Cell. 2015;162(5):1051–1065. doi: 10.1016/j.cell.2015.07.048 26300125 PMC4556133

[47] Mavrommatis E, Fish EN, Platanias LC. The schlafen family of proteins and their regulation by interferons. Journal of Interferon & Cytokine Research. 2013;33(4):206–210. doi: 10.1089/jir.2012.0133 23570387 PMC3624771

[48] Leek JT, Storey JD. Capturing heterogeneity in gene expression studies by Surrogate Variable Analysis. PLoS Genetics. 2007;3(9):e161. doi: 10.1371/journal.pgen.0030161 17907809 PMC1994707

[49] Bowden J, Davey Smith G, Burgess S. Mendelian randomization with invalid instruments: effect estimation and bias detection through Egger regression. International Journal of Epidemiology. 2015;44(2):512–525. doi: 10.1093/ije/dyv080 26050253 PMC4469799

[50] Bowden J, Davey Smith G, Haycock PC, Burgess S. Consistent Estimation in Mendelian Randomization with Some Invalid Instruments Using a Weighted Median Estimator. Genetic Epidemiology. 2016;40(4):304–314. doi: 10.1002/gepi.21965 27061298 PMC4849733

[51] Verbanck M, Chen CY, Neale B, Do R. Detection of widespread horizontal pleiotropy in causal relationships inferred from Mendelian randomization between complex traits and diseases. Nature Genetics. 2018;50(5):693–698. doi: 10.1038/s41588-018-0099-7 29686387 PMC6083837

[52] Morrison J, Knoblauch N, Marcus JH, Stephens M, He X. Mendelian randomization accounting for correlated and uncorrelated pleiotropic effects using genome-wide summary statistics. Nature Genetics. 2020;52(7):740–747. doi: 10.1038/s41588-020-0631-4 32451458 PMC7343608

[53] Giambartolomei C, Vukcevic D, Schadt EE, Franke L, Hingorani AD, Wallace C, et al. Bayesian Test for Colocalisation between Pairs of Genetic Association Studies Using Summary Statistics. PLOS Genetics. 2014;10(5):1–15. doi: 10.1371/journal.pgen.1004383 24830394 PMC4022491

[54] Pickrell JK, Berisa T, Liu JZ, Ségurel L, Tung JY, Hinds DA. Detection and interpretation of shared genetic influences on 42 human traits. Nature Genetics. 2016;48(7):709–717. doi: 10.1038/ng.3570 27182965 PMC5207801

[55] Hormozdiari F, van de Bunt M, Segrè AV, Li X, Joo JWJ, Bilow M, et al. Colocalization of GWAS and eQTL Signals Detects Target Genes. The American Journal of Human Genetics. 2016;99(6). doi: 10.1016/j.ajhg.2016.10.003 27866706 PMC5142122

[56] Foley CN, Staley JR, Breen PG, Sun BB, Kirk PDW, Burgess S, et al. A fast and efficient colocalization algorithm for identifying shared genetic risk factors across multiple traits. Nature Communications. 2021;12(1). doi: 10.1038/s41467-020-20885-8 33536417 PMC7858636

[57] Wallace C. A more accurate method for colocalisation analysis allowing for multiple causal variants. PLoS Genetics. 2021;17(9):e1009440. doi: 10.1371/journal.pgen.1009440 34587156 PMC8504726

[58] Breiman L. Bagging predictors. Machine Learning. 1996;24(2):123–140. doi: 10.1007/BF00058655

[59] Valdar W, Holmes CC, Mott R, Flint J. Mapping in structured populations by resample model averaging. Genetics. 2009;182(4):1263–1277. doi: 10.1534/genetics.109.100727 19474203 PMC2728864

[60] Meinshausen N, Bühlmann P. Stability selection. Journal of the Royal Statistical Society: Series B (Statistical Methodology). 2010;72(4):417–473. doi: 10.1111/j.1467-9868.2010.00740.x

[61] Valdar W, Sabourin J, Nobel A, Holmes CC. Reprioritizing genetic associations in hit regions using LASSO-based resample model averaging. Genetic Epidemiology. 2012;36(5):451–462. doi: 10.1002/gepi.21639 22549815 PMC3470705

[62] Sabourin J, Nobel AB, Valdar W. Fine-mapping additive and dominant SNP effects using group-LASSO and fractional resample model averaging. Genetic Epidemiology. 2015;39(2):77–88. doi: 10.1002/gepi.21869 25417853 PMC4314429

[63] Hayes AF, Preacher KJ. Statistical mediation analysis with a multicategorical independent variable. British Journal of Mathematical and Statistical Psychology. 2014;67(3):451–470. doi: 10.1111/bmsp.12028 24188158

[64] James LR, Brett JM. Mediators, moderators, and tests for mediation. Journal of Applied Psychology. 1984;69(2):307–321. doi: 10.1037/0021-9010.69.2.307

[65] Muller D, Judd CM, Yzerbyt VY. When moderation is mediated and mediation is moderated. Journal of Personality and Social Psychology. 2005;89(6):852–863. doi: 10.1037/0022-3514.89.6.852 16393020

[66] George EI, McCulloch RE. Variable selection via Gibbs sampling. Journal of the American Statistical Association. 1993;88(423):881–889. doi: 10.1080/01621459.1993.10476353

[67] Otter T, Pachali MJ, Mayer S, Landwehr J. Causal inference using mediation analysis or instrumental variables — full mediation in the absence of conditional independence. Marketing ZFP—Journal of Research and Management. 2018;40(2):41–57.

[68] Hayes AF, Scharkow M. The relative trustworthiness of inferential tests of the indirect effect in statistical mediation analysis. Psychological Science. 2013;24(10):1918–1927. doi: 10.1177/0956797613480187 23955356

[69] Hayes AF. Beyond Baron and Kenny: statistical mediation analysis in the new millennium. Communication Monographs. 2009;76(4):408–420. doi: 10.1080/03637750903310360

[70] Fritz MS, MacKinnon DP. Required Sample Size to Detect the Mediated Effect. Psychological Science. 2007;18(3):233–239. doi: 10.1111/j.1467-9280.2007.01882.x 17444920 PMC2843527

[71] Rucker DD, Preacher KJ, Tormala ZL, Petty RE. Mediation analysis in social psychology: current practices and new recommendations. Social and Personality Psychology Compass. 2011;5(6):359–371. doi: 10.1111/j.1751-9004.2011.00355.x

[72] Glover F, Taillard E, Taillard E. A user’s guide to tabu search. Annals of Operations Research. 1993;41(1):1–28. doi: 10.1007/BF02078647

[73] Keele GR, Crouse WL, Kelada SNP, Valdar W. Determinants of QTL mapping power in the realized Collaborative Cross. G3 (Bethesda, Md). 2019;9(May):459966. 10.1534/g3.119.400194 PMC6505132 30914424

[74] Gatti DM, Simecek P, Somes L, Jeffrey CT, Vincent MJ, Choi K, et al. The effects of sex and diet on physiology and liver gene expression in Diversity Outbred mice. bioRxiv. 2017. doi: 10.1534/genetics.116.198051 28592500 PMC5499176

[75] Beasley TM, Erickson S, Allison DB. Rank-based inverse normal transformations are increasingly used, but are they merited? Behavior Genetics. 2009;39(5):580–595. doi: 10.1007/s10519-009-9281-0 19526352 PMC2921808

[76] R/qtl2: software for mapping quantitative trait loci with high-dimensional data and multiparent populations. Genetics. 2019;211(2):495–502. doi: 10.1534/genetics.118.301595 30591514 PMC6366910

[77] van de Geijn B, McVicker G, Gilad Y, Pritchard JK. WASP: allele-specific software for robust molecular quantitative trait locus discovery. Nature Methods. 2015;12(11):1061–1063. doi: 10.1038/nmeth.3582 26366987 PMC4626402

[78] R Core Team. R: a language and environment for statistical computing; 2022. Available from: https://www.R-project.org/.

