# Supplementary material for: A Bayesian model selection approach to mediation analysis
Source: PLoS Genet. 2022 May 9;18(5):e1010184. doi: 10.1371/journal.pgen.1010184 (PMC9129027; doi:10.1371/journal.pgen.1010184)
Supplement: S2 Fig — Data for 200 individuals were simulated according to (a) reactive partial mediation and (b) reactive complete mediation models from a balanced bi-allelic SNP. DAGs indicate the model used to simulate the data. Heat maps represent the mean posterior probability associated with each inferred model for a range of fixed settings of the model parameters as indicated on x- and y-axes, each simulated 100 times. Bayesian model selection was performed using the (left) default effect size priors (50% for a, b, and c) and (right) empirical effect size priors. Model priors were varied, represented as the rows within an individual panel. Empty squares represent posterior model categories not evaluated based on the set of allowable models encoded in the model priors. See S1 Fig for results from non-reactive model simulations. (PDF) [file pgen.1010184.s002.pdf]

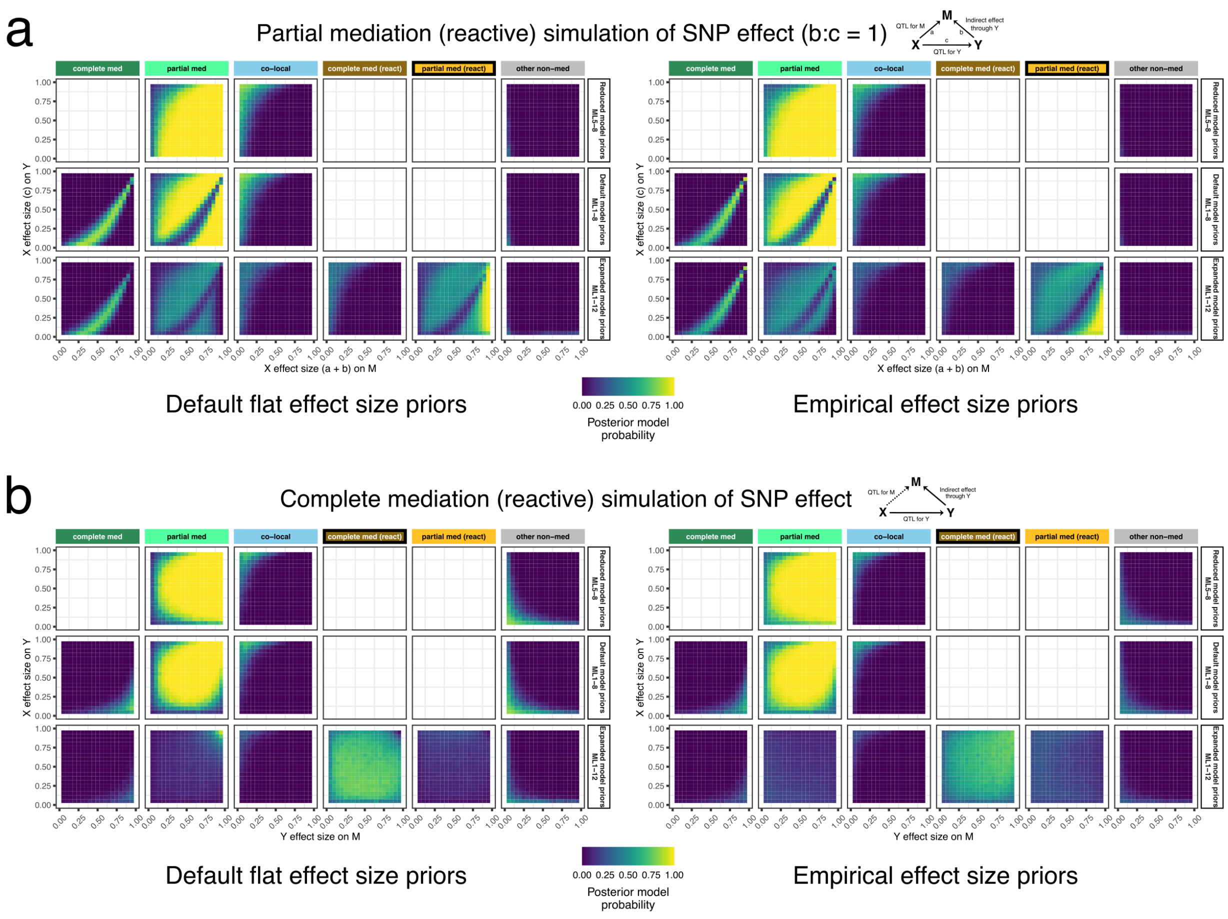

S2 Fig. Performance of Bayesian model selection in simulated QTL data from reactive models across varying priors for effect size and allowable models. Data for 200 individuals were simulated according to (a) reactive partial mediation and (b) reactive complete mediation models from a balanced bi-allelic SNP. DAGs indicate the model used to simulate the data. Heat maps represent the mean posterior probability associated with each inferred model for a range of fixed settings of the model parameters as indicated on x- and y-axes, each simulated 100 times. Bayesian model selection was performed using the (left) default effect size priors (50% for a, b, and c) and (right) empirical effect size priors. Model priors were varied, represented as the rows within an individual panel. Empty squares represent posterior model categories not evaluated based on the set of allowable models encoded in the model priors. See S1 Fig for results from non-reactive model simulations.
