# Supplementary material for: A Bayesian model selection approach to mediation analysis
Source: PLoS Genet. 2022 May 9;18(5):e1010184. doi: 10.1371/journal.pgen.1010184 (PMC9129027; doi:10.1371/journal.pgen.1010184)
Supplement: S3 Fig — Data for 200 individuals were simulated according to (a) co-local, (b) partial mediation, and (c) complete mediation. DAGs indicate the model used to simulate the data. Heat maps for Bayesian model selection represent the mean posterior probability associated with each inferred model for a range of fixed settings of the model parameters as indicated on x- and y-axes, each simulated 100 times. Default prior settings were used. Heat maps for Bayesian network analysis represent the best bnlearn model probability across 100 simulations. See S1 and S2 Figs for Bayesian model selection results using empirical effect size priors and non-default model priors, including reactive. See S4 and S5 Figs for similar results from Bayesian network analysis. (PDF) [file pgen.1010184.s003.pdf]

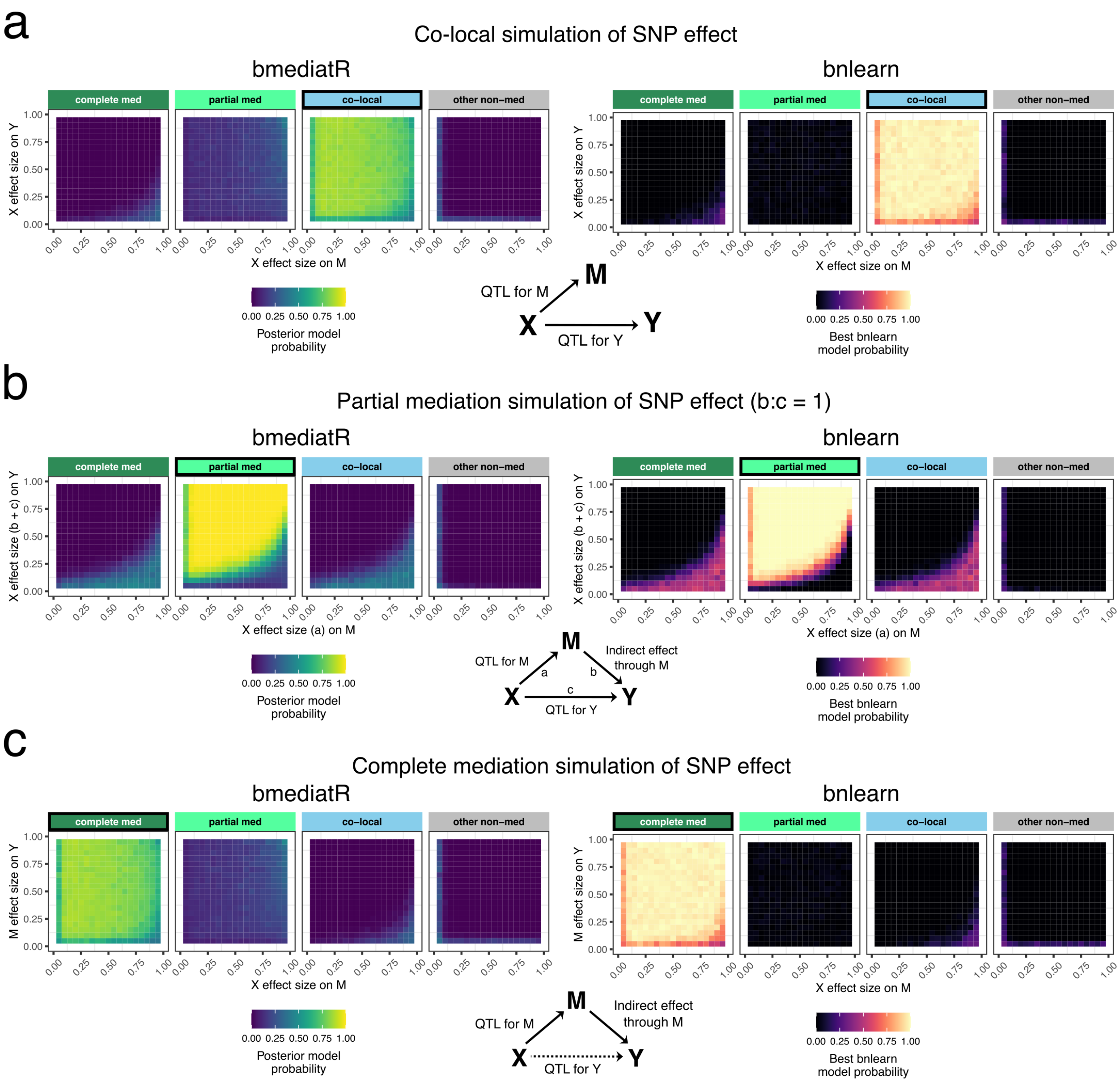

S3 Fig. Performance of Bayesian model selection (bmediatR) compared with a Bayesian network analysis (bnlearn) in simulated data with a binary exogenous variable. Data for 200 individuals were simulated according to (a) co-local, (b) partial mediation, and (c) complete mediation. DAGs indicate the model used to simulate the data. Heat maps for Bayesian model selection represent the mean posterior probability associated with each inferred model for a range of fixed settings of the model parameters as indicated on x- and y-axes, each simulated 100 times. Default prior settings were used. Heat maps for Bayesian network analysis represent the best bnlearn model probability across 100 simulations. See S1 and S2 Figs for Bayesian model selection results using empirical effect size priors and non-default model priors, including reactive. See S4 and S5 Figs for similar results from Bayesian network analysis.
