# Supplementary material for: A Bayesian model selection approach to mediation analysis
Source: PLoS Genet. 2022 May 9;18(5):e1010184. doi: 10.1371/journal.pgen.1010184 (PMC9129027; doi:10.1371/journal.pgen.1010184)
Supplement: S5 Fig — Data for 200 individuals were simulated according to (a) reactive partial mediation and (b) reactive complete mediation models from a balanced bi-allelic SNP. DAGs indicate the model used to simulate the data. Heat maps for Bayesian network analysis represent the best bnlearn model probability across 100 simulations for a range of fixed settings of the model parameters as indicated on x- and y-axes. Heat maps for the Sobel test and IV regression represent false positive probability for co-local simulations and power for mediation simulations. Heat maps for LOD drop represent mean LOD drop, scaled to the proportion of the simulated QTL’s LOD score. Model priors were varied, represented as the rows within an individual panel. Empty squares represent posterior model categories not evaluated based on the set of allowable models encoded in the model priors. See S4 Fig for results from non-reactive model simulations. (PDF) [file pgen.1010184.s005.pdf]

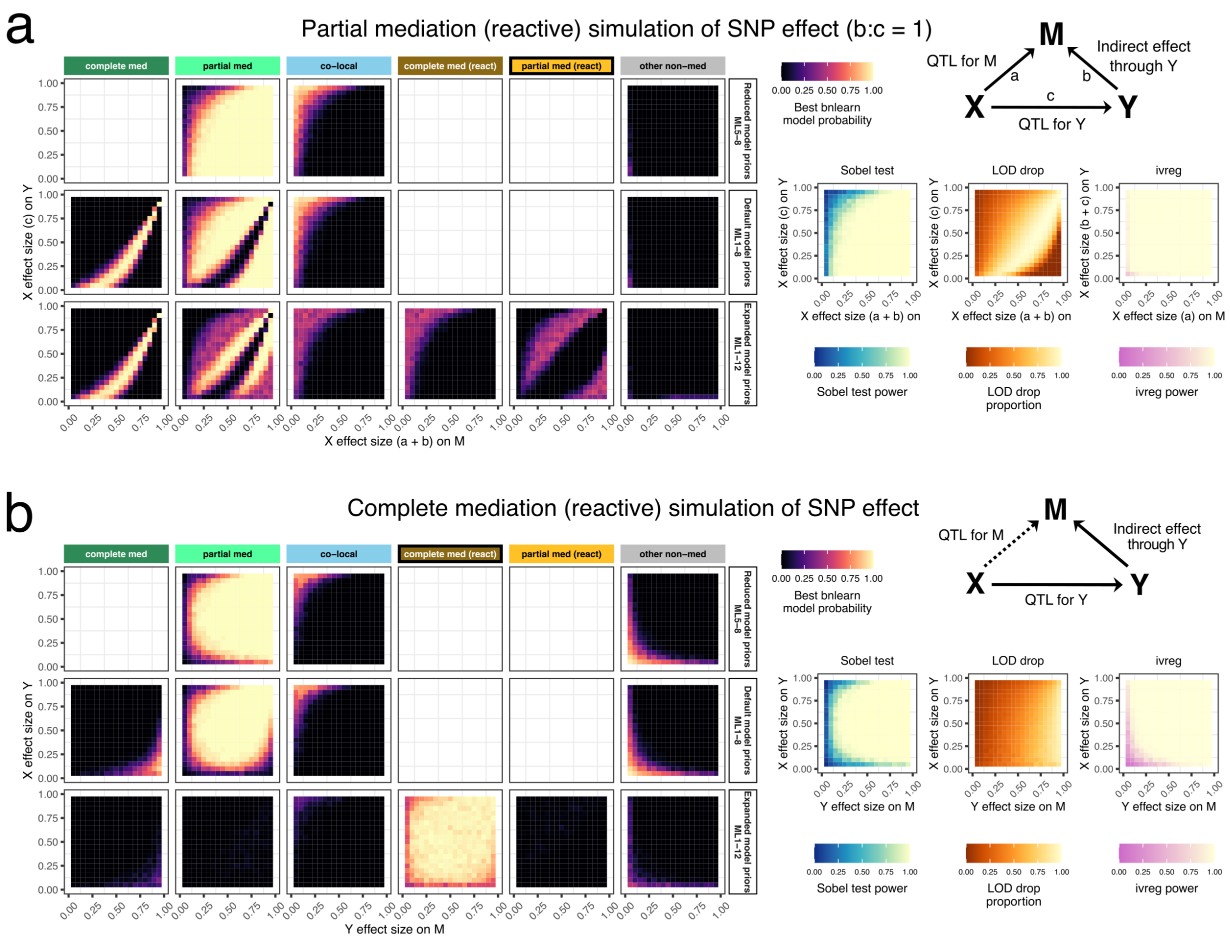

S5 Fig. Performance of Bayesian network analysis, Sobel test, LOD drop, and IV regression in simulated QTL data from reactive models. Data for 200 individuals were simulated according to (a) reactive partial mediation and (b) reactive complete mediation models from a balanced bi-allelic SNP. DAGs indicate the model used to simulate the data. Heat maps for Bayesian network analysis represent the best bnlearn model probability across 100 simulations for a range of fixed settings of the model parameters as indicated on x- and y-axes. Heat maps for the Sobel test and IV regression represent false positive probability for co-local simulations and power for mediation simulations. Heat maps for LOD drop represent mean LOD drop, scaled to the proportion of the simulated QTL's LOD score. Model priors were varied, represented as the rows within an individual panel. Empty squares represent posterior model categories not evaluated based on the set of allowable models encoded in the model priors. See S4 Fig for results from non-reactive model simulations.
