# Supplementary material for: A Bayesian model selection approach to mediation analysis
Source: PLoS Genet. 2022 May 9;18(5):e1010184. doi: 10.1371/journal.pgen.1010184 (PMC9129027; doi:10.1371/journal.pgen.1010184)
Supplement: S6 Fig — (a) Data for 200 individuals were simulated according to partial mediation. The DAG represents the partial mediation model used to simulate the data. Heat maps for Bayesian model selection represent the mean posterior probability associated with each inferred model for a range of fixed settings of the model parameters as indicated on x- and y-axes, each simulated 100 times. Default prior settings were used. Heat maps for Bayesian network analysis represent the best bnlearn model probability, across 100 simulations. Two effect size settings are marked and explored further: (b) + and (c) ×. For each scenario (b-c), bmediatR returned posterior model probabilities (left) and bnlearn returned best models (right) for 10,000 simulations. Best models from bnlearn are represented as stacked bar plots for each model, with solid bars indicating the number of simulations for which the model was selected as best model and transparent bars indicate the number of simulations for which it was not selected. (d) Posterior model probabilities for a simulated mediator that bnlearn classified correctly as partial (left) and incorrectly as co-local (right). (PDF) [file pgen.1010184.s006.pdf]

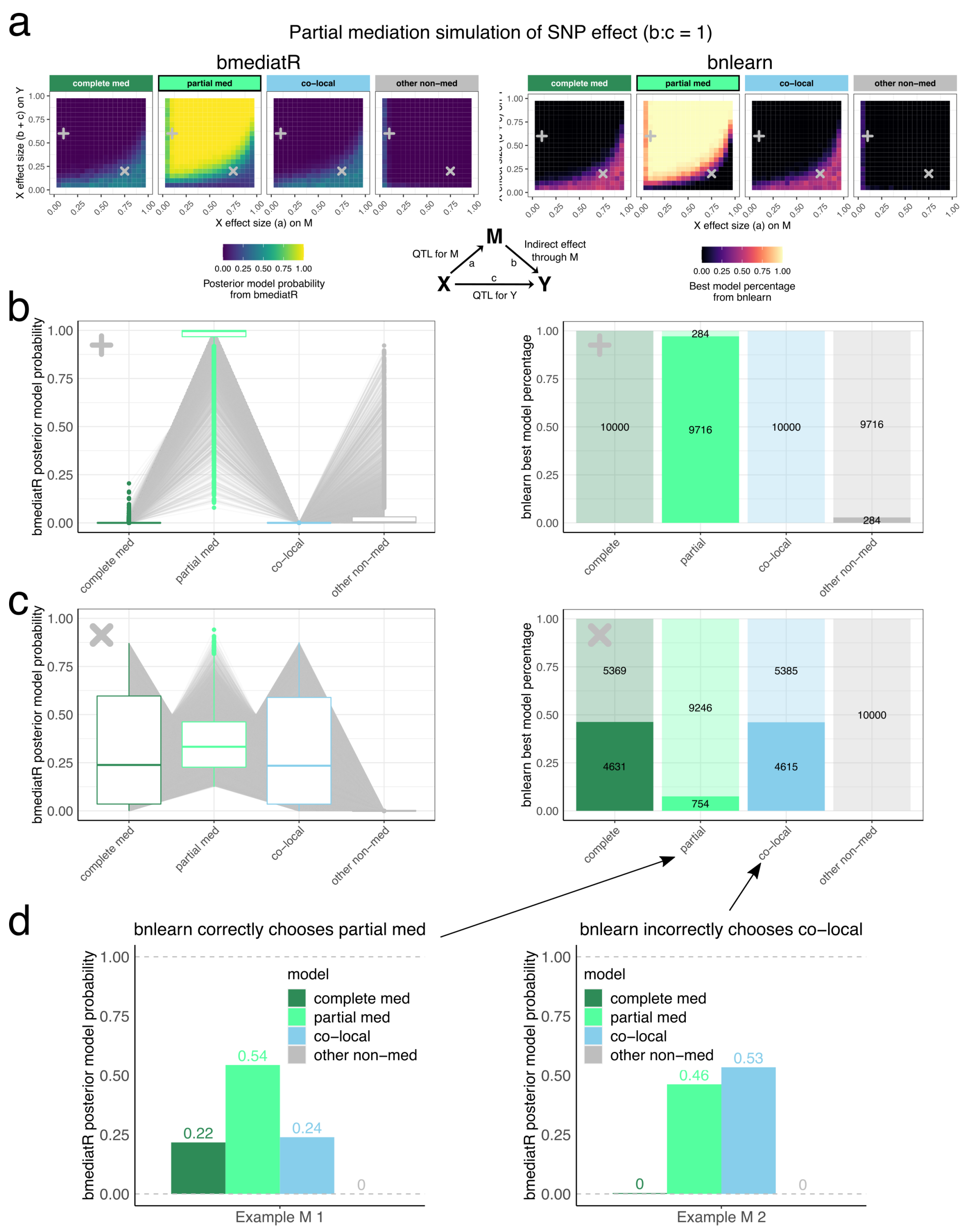

S6 Fig. Example scenarios comparing inference from Bayesian model selection (bmediatR) to a Bayesian network analysis (bnlearn) in simulated data with a binary exogenous variable. (a) Data for 200 individuals were simulated according to partial mediation. The DAG represents the partial mediation model used to simulate the data. Heat maps for Bayesian model selection represent the mean posterior probability associated with each inferred model for a range of fixed settings of the model parameters as indicated on x- and y-axes, each simulated 100 times. Default prior settings were used. Heat maps for Bayesian network analysis represent the best bnlearn model probability, across 100 simulations. Two effect size settings are marked and explored further: (b) + and (c) ×. For each scenario (b-c), bmediatR returned posterior model probabilities (left) and bnlearn returned best models (right) for 10,000 simulations. Best models from bnlearn are represented as stacked bar plots for each model, with solid bars indicating the number of simulations for which the model was selected as best model and transparent bars indicate the number of simulations for which it was not selected. (d) Posterior model probabilities for a simulated mediator that bnlearn classified correctly as partial (left) and incorrectly as co-local (right).
