# Supplementary material for: A Bayesian model selection approach to mediation analysis
Source: PLoS Genet. 2022 May 9;18(5):e1010184. doi: 10.1371/journal.pgen.1010184 (PMC9129027; doi:10.1371/journal.pgen.1010184)
Supplement: S7 Fig — True positive rates (power) and false positive rates were estimated from 5,000 simulations of 24 individuals according to a balanced bi-allelic variant X. Results are shown for data simulated with both (a-c) small genetic effects (X → M: 10%, M → Y: 5%, X → Y: 5%) and (b-d) large genetic effects (X → M: 50%, M → Y: 25%, X → Y: 25%). Diagonal dashed line is included for reference, representing a classifier with no ability to distinguish complete mediation from co-local or non-mediation. See Fig 3 for methods’ performance in distinguishing complete mediation from co-local and non-mediation. (PDF) [file pgen.1010184.s007.pdf]

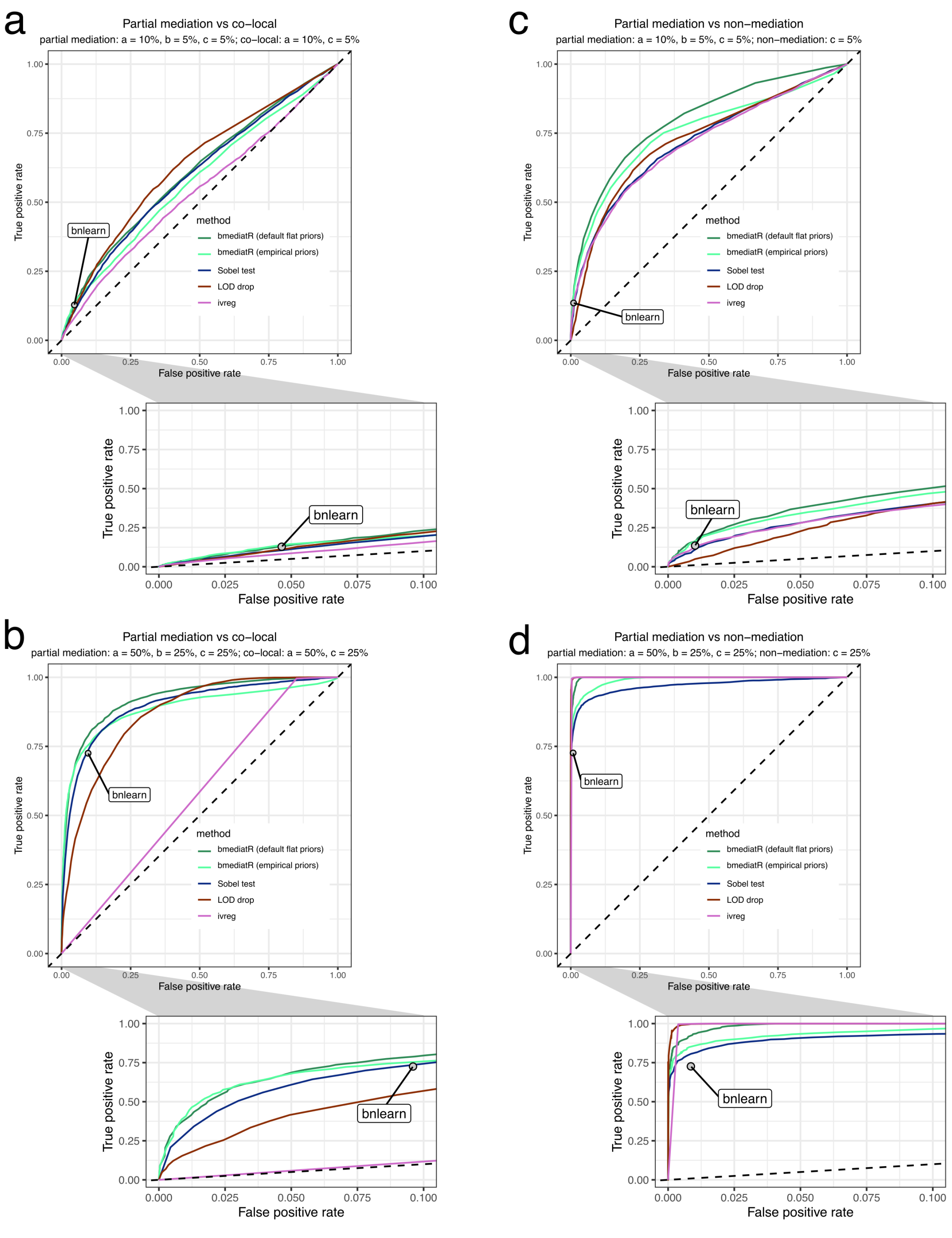

S7 Fig. Performance of Bayesian model selection compared with other methods in distinguishing partial mediation from (a-b) co-local and (c-d) non-mediation. True positive rates (power) and false positive rates were estimated from 5,000 simulations of 24 individuals according to a balanced bi-allelic variant X. Results are shown for data simulated with both (a-c) small genetic effects (X $\rightarrow$ M: 10%, M $\rightarrow$ Y: 5%, X $\rightarrow$ Y: 5%) and (b-d) large genetic effects (X $\rightarrow$ M: 50%, M $\rightarrow$ Y: 25%, X $\rightarrow$ Y: 25%). Diagonal dashed line is included for reference, representing a classifier with no ability to distinguish complete mediation from co-local or non-mediation. See Fig 3 for methods' performance in distinguishing complete mediation from co-local and non-mediation.
