# Supplementary material for: A Bayesian model selection approach to mediation analysis
Source: PLoS Genet. 2022 May 9;18(5):e1010184. doi: 10.1371/journal.pgen.1010184 (PMC9129027; doi:10.1371/journal.pgen.1010184)
Supplement: S8 Fig — The allelic series were modeled with TIMBR. (a) Posterior haplotype effects and allelic series for the Snx4 distal pQTL and the local pQTL for its mediator, Snx7. Haploytpe effects are represented as histograms of the posterior samples. Allelic series are represented as circos plots where the opacity of the links represent how often TIMBR assigned founder haplotypes to the same functional allele. (b) Posterior haplotype effects and allelic series for the Tubg1 and Tubgcp2 distal pQTL and the local pQTL for their candidate mediators, Tubgcp3 and Naxd. The posterior expected number of functional alleles, k, is included for each pQTL. (PDF) [file pgen.1010184.s008.pdf]

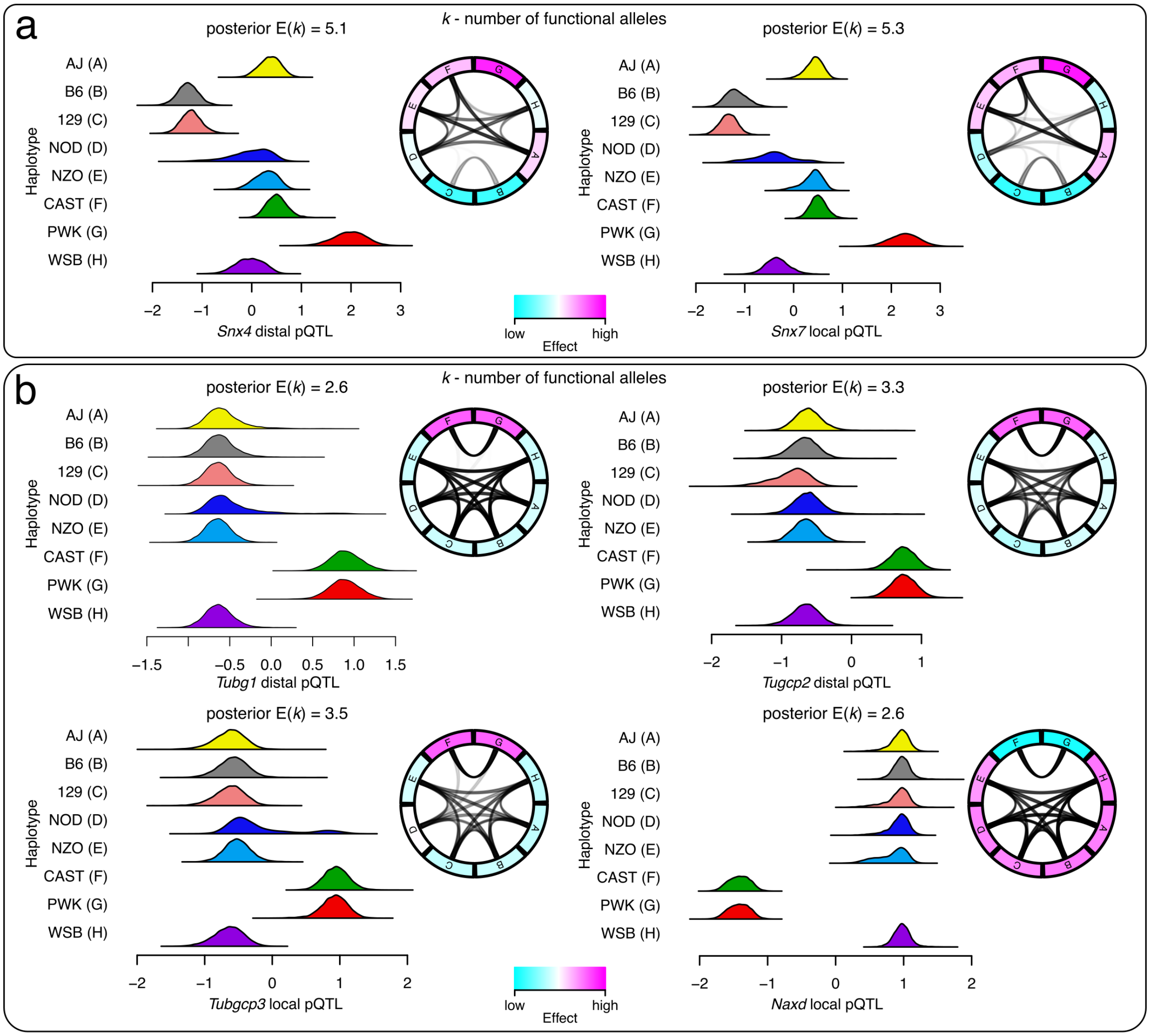

S8 Fig. Modeling the allelic series of pQTL for *Snx4*, *Tubg1*, and related genes identified through mediation. The allelic series were modeled with TIMBR. (a) Posterior haplotype effects and allelic series for the *Snx4* distal pQTL and the local pQTL for its mediator, *Snx7*. Haploytpe effects are represented as histograms of the posterior samples. Allelic series are represented as circos plots where the opacity of the links represent how often TIMBR assigned founder haplotypes to the same functional allele. (b) Posterior haplotype effects and allelic series for the *Tubg1* and *Tubgcp2* distal pQTL and the local pQTL for their candidate mediators, *Tubgcp3* and *Naxd*. The posterior expected number of functional alleles, k, is included for each pQTL.
