# Supplementary material for: A Bayesian model selection approach to mediation analysis
Source: PLoS Genet. 2022 May 9;18(5):e1010184. doi: 10.1371/journal.pgen.1010184 (PMC9129027; doi:10.1371/journal.pgen.1010184)
Supplement: S1 Table — (PDF) [file pgen.1010184.s009.pdf]

S1 Table: Features of the evaluated causal inference methods

Method	Multi-state X	Complete vs partial	Mediation vs non-mediation	Priors	Summary
bmediatR	x	x	x	X[b]	Model posterior probabilities
Sobel test			x		Indirect effect *p*-value
LOD drop	x		x		Change in LOD score
ivreg	x		x		Causal effect *p*-value[d]
bnlearn	x[a]	x	x	X[c]	Best model (0/1)

[a]Selects from columns of X matrix, which could be appealing for multiple genetic variants but unappealing for multi-state genotypes.
[b]Effect size priors (default: 50% for a, b, and c) and model priors (default: equal probabilities, excluding reactive models).
[c]Model priors (limited to fixing or excluding relationships). Graph priors have similar effect to effect size priors of bmediatR.
[d]Assumes complete mediation (no direct effect of X on Y).
