# Supplementary material for: A Bayesian model selection approach to mediation analysis
Source: PLoS Genet. 2022 May 9;18(5):e1010184. doi: 10.1371/journal.pgen.1010184 (PMC9129027; doi:10.1371/journal.pgen.1010184)
Supplement: S1 Text — (PDF) [file pgen.1010184.s010.pdf]

S1 Appendix: Joint likelihood for causal models in the reactive mode

For causal models describing reverse causality between M and Y, that is, the reactive models $\boldsymbol{\theta} = (\theta_a, \theta_b = *, \theta_c)$, the roles of M and Y are switched relative to the other models, so that the conditional joint likelihood $\mathrm{p}(\mathbf{y}, \mathbf{m}|\boldsymbol{\theta})$ in these case is

$$\mathbf{m}|\mathbf{y}, \theta_a, \theta_b = *, \mu_{\mathbf{m}}, \boldsymbol{\alpha}_{\mathbf{m}}, \boldsymbol{\beta}_a, \beta_b, \sigma_{\mathbf{m}} \sim \mathrm{N}(\mu_{\mathbf{m}}\mathbf{1} + \mathbf{Z}_{\mathbf{m}}\boldsymbol{\alpha}_{\mathbf{m}} + \theta_a\mathbf{X}\boldsymbol{\beta}_a + \mathbf{y}\beta_b, \sigma^2_{\mathbf{m}}\mathbf{W}^{-1}_{\mathbf{m}}),$$
$$\mathbf{y}|\theta_b = *, \theta_c, \mu_{\mathbf{y}}, \boldsymbol{\alpha}_{\mathbf{y}}, \boldsymbol{\beta}_c, \sigma_{\mathbf{y}} \sim \mathrm{N}(\mu_{\mathbf{y}}\mathbf{1} + \mathbf{Z}_{\mathbf{y}}\boldsymbol{\alpha}_{\mathbf{y}} + \theta_c\mathbf{X}\boldsymbol{\beta}_c, \sigma^2_{\mathbf{y}}\mathbf{W}^{-1}_{\mathbf{y}}),$$

where β_b is now the scalar effect of $\mathbf{y}$ on $\mathbf{m}$. Prior distributions for all variables are unchanged (except for $\boldsymbol{\theta}$). When $\theta_b = *$, the marginal joint likelihood function is given by:

$$\mathbf{m}|\mathbf{y}, \theta_a, \theta_b = * \sim \mathrm{t}_{\kappa_{\mathbf{m}}}(\mathbf{0}, \lambda_{\mathbf{m}}[\mathbf{W}^{-1} + \mathcal{X}_{\mathbf{m}}\mathbf{V}_{\mathbf{m}}\mathcal{X}^T_{\mathbf{m}}])$$
$$\mathbf{y}|\theta_b = *, \theta_c \sim \mathrm{t}_{\kappa_{\mathbf{y}}}(\mathbf{0}, \lambda_{\mathbf{y}}[\mathbf{W}^{-1} + \mathcal{X}_{\mathbf{y}}\mathbf{V}_{\mathbf{y}}\mathcal{X}^T_{\mathbf{y}}])$$

$$\mathcal{X}_{\mathbf{m}} = \begin{bmatrix}\mathbf{1} & \mathbf{Z}_{\mathbf{m}} & \theta_a\mathbf{X} & \mathbf{y}\end{bmatrix} \qquad \mathbf{V}_{\mathbf{m}} = \begin{bmatrix}\tau^2_\mu & \mathbf{0} & \mathbf{0} & 0\\ \mathbf{0} & \tau^2_{\mathbf{Z}}\mathbf{I} & \mathbf{0} & \mathbf{0}\\ \mathbf{0} & \mathbf{0} & \phi^2_a\mathbf{I} & \mathbf{0}\\ 0 & \mathbf{0} & \mathbf{0} & \phi^2_b\end{bmatrix}$$

$$\mathcal{X}_{\mathbf{y}} = \begin{bmatrix}\mathbf{1} & \mathbf{Z}_{\mathbf{y}} & \theta_c\mathbf{X}\end{bmatrix} \qquad \mathbf{V}_{\mathbf{y}} = \begin{bmatrix}\tau^2_\mu & \mathbf{0} & \mathbf{0}\\ \mathbf{0} & \tau^2_{\mathbf{Z}}\mathbf{I} & \mathbf{0}\\ \mathbf{0} & \mathbf{0} & \phi^2_c\mathbf{I}\end{bmatrix}$$

Hyperparameters for $\boldsymbol{\kappa}, \boldsymbol{\lambda}, \boldsymbol{\tau}_\mu, \boldsymbol{\tau}_{\mathbf{Z}}$, and $\boldsymbol{\phi}^2$ are unchanged.
